# A large intragenic deletion in the *CLCN1* gene causes Hereditary Myotonia in pigs

**DOI:** 10.1038/s41598-019-51286-7

**Published:** 2019-10-30

**Authors:** C. E. T. Araújo, C. M. C. Oliveira, J. D. Barbosa, J. P. Oliveira-Filho, L. A. L. Resende, P. R. Badial, J. P. Araujo-Junior, M. E. McCue, A. S. Borges

**Affiliations:** 1 São Paulo State University (UNESP), School of Veterinary Medicine and Animal Science, Botucatu, São Paulo Brazil; 2 Instituto de Medicina Veterinária, Universidade Federal do Pará, Campus Castanhal, PA Brazil; 3 São Paulo State University (UNESP), Medical School, Botucatu, Brazil; 4 Department of Pathobiology and Population Medicine, College of Veterinary Medicine, Mississippi State University, Starkville, MS USA; 5 São Paulo State University (UNESP), Institute of Bioscience, Botucatu, Brazil; 6 College of Veterinary Medicine, University of Minnesota, St Paul, Minnesota 55108 USA

**Keywords:** Mutation, Neurophysiology

## Abstract

Mutations in the *CLCN1* gene are the primary cause of non-dystrophic Hereditary Myotonia in several animal species. However, there are no reports of Hereditary Myotonia in pigs to date. Therefore, the objective of the present study was to characterize the clinical and molecular findings of Hereditary Myotonia in an inbred pedigree. The clinical, electromyographic, histopathological, and molecular findings were evaluated. Clinically affected pigs presented non-dystrophic recessive Hereditary Myotonia. Nucleotide sequence analysis of the entire coding region of the *CLCN1* gene revealed the absence of the exons 15 and 16 in myotonic animals. Analysis of the genomic region flanking the deletion unveiled a large intragenic deletion of 4,165 nucleotides. Interestingly, non-related, non-myotonic pigs expressed transcriptional levels of an alternate transcript (i.e., X2) that was identical to the deleted X1 transcript of myotonic pigs. All myotonic pigs and their progenitors were homozygous recessive and heterozygous, respectively, for the 4,165-nucleotide deletion. This is the first study reporting Hereditary Myotonia in pigs and characterizing its clinical and molecular findings. Moreover, to the best of our knowledge, Hereditary Myotonia has never been associated with a genomic deletion in the *CLCN1* gene in any other species.

## Introduction

Myotonia is a medical term describing a delayed relaxation of the skeletal muscle after voluntary contraction, electrical or mechanical stimuli^1,2^. Disorders exhibiting myotonia are known as myotonic diseases and can be classified in non-dystrophic and dystrophic^3^. The leading causes of the former (i.e., Myotonia Congenita or Hereditary Myotonia) are mutations in the *CLCN1* gene inherited in either an autosomal dominant or recessive mode^4–6^. Hereditary Myotonia has extensively been reported in humans^7–13^, and it has been associated with more than 200 different mutations affecting the *CLCN1* gene^14^. This disease has also been associated with mutations in the *CLCN1* gene in rats^15^, goats^16^, dogs^17,18^, horses^19^, buffalo^20^, cats^21^, and sheep^22^. It is clinically characterized by muscle stiffness after stimuli, muscle hypertrophy (mainly observed in the epaxial and appendappendicular muscles), and warm-up effect^7–13,15–21^. Humans and animals affected with Hereditary Myotonia, even with the same causative mutation, can present variable intensity of the clinical signs^17,18,20,21,23–25^.

The *CLCN1* gene encodes for the chloride channel protein 1 (CLC-1), which is the most abundant chloride channel protein expressed in the skeletal muscle tissue^1,26^. The CLC-1 is a plasma membrane homodimeric protein with each subunit containing a selective chloride pore region that can function independently^27–31^. Functional CLC-1 proteins are crucial to ensure the electrical stability of the muscle cells since the sarcolemmal chloride conductance at rest represents up to 85% of all ionic conductance of the sarcolemma^32,33^.

In this study, we describe for the first time the clinical and molecular characterization of a form of Hereditary Myotonia affecting pigs. The condition seems to follow an autosomal recessive inheritance pattern exhibiting classical clinical signs of Hereditary Myotonia. The disease is associated with a large intragenic deletion in the *CLCN1* gene.

## Results

### Clinical evaluation

To clinically characterize the musculoskeletal disorder affecting the studied animals, we performed physical, histopathologic, and electromyography examinations. We also carried out a pedigree analysis to evaluate the inheritance pattern of the disease.

Myotonic pigs belonged to a small rural property located in the state of Pará in the North Region of Brazil. All myotonic animals were mixed-breed pigs not belonging to any commercial lineages and comprised a group of animals exhibiting high inbreeding rate.

**Figure 1:**
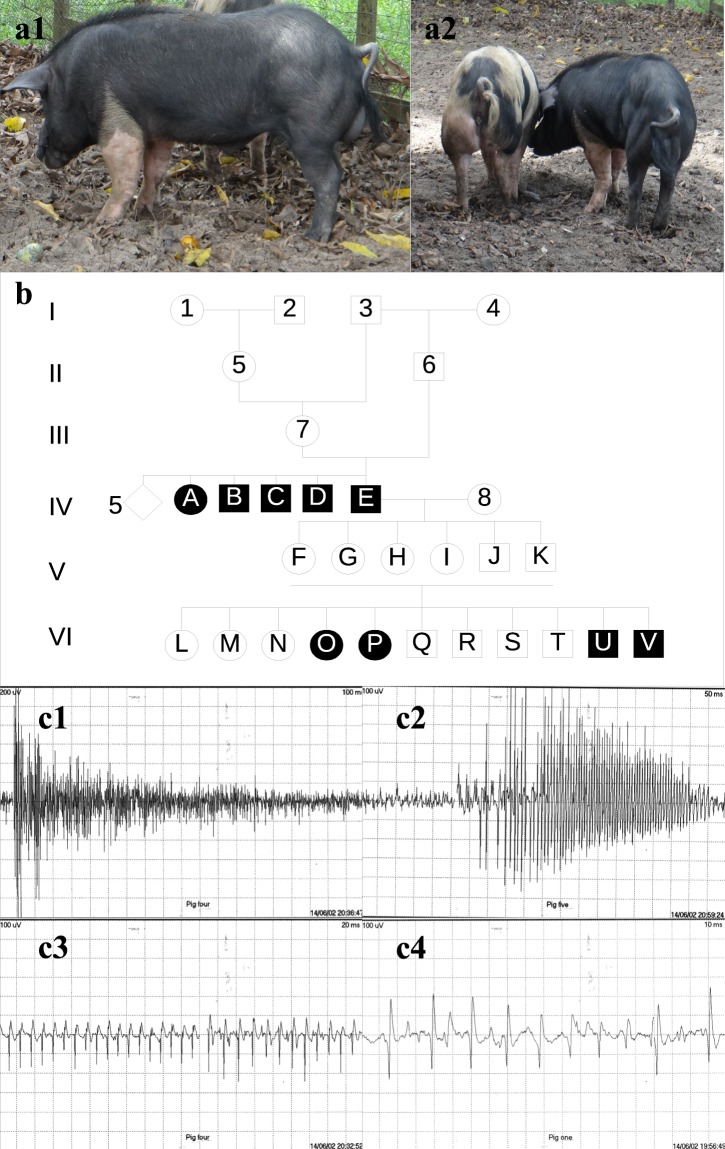
(**a**) Myotonic pigs. a1- Myotonic pig E, lateral view demonstrating muscular hypertrophy in the left pelvic limb. a2 **–** Myotonic pigs A (left) and E (right), posterior view of two animals affected by Hereditary Myotonia; note the muscular hypertrophy in the pelvic limb. (**b)** Pedigree analysis of the studied family of pigs. Note that the common ancestor (animal 3) is the progenitor of pigs (animals 6 and 7) producing myotonic animals. Roman numerals indicate the different generations, Arabic numerals indicate non-studied related animals, capital letters indicate studied animals, and the number 8 indicates an unrelated Large White sow. Squares, circles, and rhombus represent males, females, and unknown sex, respectively. Shaded and unshaded geometrical shapes indicate myotonic and non-myotonic animals. Number 5, outside the rhombus indicates the number of non-myotonic pigs in generation IV. (**c**) Electromyography of affected animals, c1 (EMG of pig D) and c2 (EMG of pig E) – Typical myotonic discharges that wax and wane in amplitude and frequency and produce the characteristic diver bomb sound (S1 audio) over the loudspeaker (analysis time of 100 and 50 mSec/div, respectively). c3 (EMG of pig D) – Sustained run of positive waves with initial sharp positivity followed by a slow negative component (analysis time of 20 mSec/div). c4 (EMG of pig A) – Sustained run of negative spikes with an initial positivity, like fibrillation potentials (analysis time of 10 mSec/div).

Upon physical examination, were identified clinical signs of Hereditary Myotonia in 41% (9/22) pigs. Affected animals exhibited similar clinical signs with variable intensities, initially observed within one week of life, which progressively worsens with age advancing. Clinical signs included myotonia, muscle hypertrophy, and stiffness. Muscle hypertrophy affected primarily epaxial and proximal muscles of the limbs, in particular, the gluteus, semimembranosus, and semitendinosus muscles (Fig. 1a). Muscle stiffness was observed in all four limbs, but it was more pronounced in the pelvic limbs. Percussion of the triceps and semitendinosus muscles efficiently elicited a sustained local muscular contraction (i.e., myotonic dimple) lasting several seconds. Affected pigs exhibited different muscle stiffness intensities and variable startle responses upon stimulation. The clinical presentation of each animal was classified in either mild, moderate, or intense according to the combined score of muscle hypertrophy, muscle stiffness, and startle response (Table 1 and S1 Table). Three animals exhibited an intense clinical phenotype with immediate startle response and exaggerated muscular rigidity. The startle response in those animals was easily triggered upon minimal stimulation leading to recumbency, especially when startled after rest periods (S1 and S2 video). Five animals presented a less severe startle response accompanied by intense muscle stiffness without recumbency. One myotonic pig exhibited a mild clinical phenotype with slight muscle stiffness and absence of startle response. The frequency of myotonic episodes diminished after exercise, indicating a warm-up phenomenon. Besides evident muscular clinical signs, no other clinical abnormalities were observed.

**Table 1:** Severity of Hereditary myotonia affecting pigs, intensity of the main clinical signs, and the respective affected animals.

Clinical myotonia severity scale	Muscle hypertrophy	Muscle stiffness	*Startle response/ recumbency*	Animals
Absent	Absent	Absent	Absent	All control and heterozygous animals.
Mild	Present	+	Absent	O
Moderate	Present	++	+	A, B, C, D, and E
Intense	Present	++	++	P, U, and V

Pedigree analysis suggested an autosomal recessive inheritance pattern (Fig. 1b). Generation IV was composed of ten animals, five non-myotonic pigs (information about the gender not available) and the first five myotonic pigs. Generation V pigs (four females and two males), which were all siblings, produced the generation VI, but accurate mating information was not available. This analysis allowed us to identify the common ancestor segregating the mutation and its offspring, which were progenitors of myotonic animals. Unfortunately, samples from those animals (i.e., pigs 1–7 [Fig. 1b]) were not available at the time of this study because they had already died.

Histopathologic examination (H&E) in the muscle biopsies of the myotonic animals (A, B, C, D, and E) revealed sparse hypertrophic fibers. However, the muscle fibers were regularly distributed and interspaced with normal connective tissue and no evidence of cellular degeneration.

Electromyography (EMG) examination of myotonic animals (A, C, D, and E) indicated the classical spontaneous myotonic discharges in all evaluated muscles (Fig. 1c). The characteristic sound of a dive-bomber or an accelerating or decelerating motorcycle engine was evident during the examination (S1 audio).

### Characterization of the *CLCN1* coding sequence

To investigate whether Hereditary Myotonia in pigs was associated with mutations in the *CLCN1* gene, we sequenced the entire coding region of two myotonic pigs (animals A and B) and two non-myotonic pigs (animals C1 and C2 of the control group). RT-PCR reactions carried out using cDNA samples of non-myotonic animals amplified specific products for each primer set designed, covering all *CLCN1* gene coding region, whereas reactions using cDNA samples of myotonic animals did not amplify nucleotides of exons 15 and 16, suggesting the absence of those exons in myotonic pigs.

The obtained nucleotide sequences were aligned with the *CLCN1* cDNA reference sequence (transcript variant X1 [XM_021078561.1]) revealing 3 and 12 single nucleotide polymorphisms (SNP) in the transcripts of non-myotonic and myotonic pigs, respectively (Table 2). The alignment also allowed us to identify the 348-nucleotide deletion in myotonic animals, spanning the exons 15 and 16. All SNPs detected in non-myotonic animals were synonymous. However, two out of the 12 SNPs identified in myotonic pigs were nonsynonymous (c.226 A > G and c.2759 C > T). These non-synonymous SNPs result in amino acid changes analogous to amino acids in this position in other species (Fig. S1).

**Table 2:** Single nucleotide polymorphisms identified in the *CLCN1* gene full coding sequence of non-myotonic and myotonic swine when compared to the reference sequence (XM_021078561.1).

Animal type	SNP	Amino acid change
Non-myotonic (Two control animals [C1])	c. 1077 T>Y	synonymous
	c. 1422 T>C	synonymous
	c. 1697 T>Y	synonymous
Myotonic (A and B)	c. 225 C>A	synonymous
	c. 226 A>G	Non-synonymous Met > Val
	c. 978 A>T	synonymous
	c. 1077 T>C	synonymous
	c. 1224 G>A	synonymous
	c. 1422 T>C	synonymous
	c. 1524 C>T	synonymous
	c. 2436 G>C	synonymous
	c. 2759 C>T	Non-synonymous Ala > Val
	c. 2802 A>G	synonymous
	c. 2826 G>A	synonymous
	c. 2964 A>G	synonymous

Since sequencing of the entire coding region of pigs A and B revealed the absence of exons 15 and 16, we next sought to characterize the relevant coding region of all myotonic pigs. RT-PCR reactions performed using cDNA samples and primers annealing on exons 15 and 16 did not generate any amplicons, whereas reactions carried out with a primer set flanking those exons amplified specific products of the expected size. Sequencing of PCR-generated amplicons also revealed a 348-nucleotide deletion involving exons 15 and 16 in all myotonic animals. That deletion was identical than that identified in pigs A and B.

### Characterization of the relevant genomic region of the *CLCN1* gene

**Figure 2:**
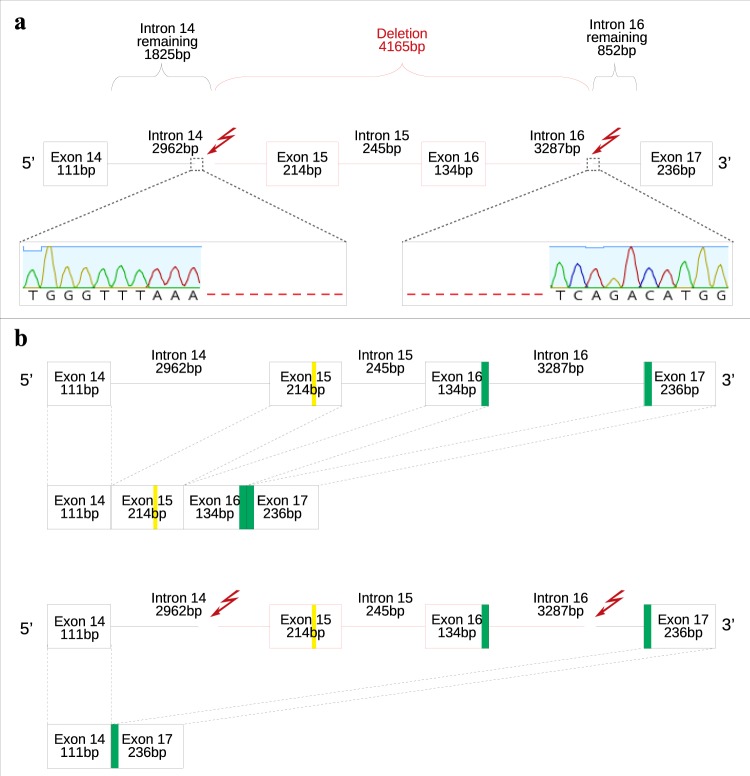
(**a**) Schematic representation of the large intragenic deletion (represented in red) (g. NC_010460.4 del 6912538_6916702) identified in the CLCN1 gene in myotonic pigs. The red arrows represent the deletion breakpoints. Electropherograms of the myotonic pigs show the 5′ and 3′ boundaries of the deletion junction. Red hyphens represent the DNA deleted fragment. (**b**) Schematic representation of CLCN1 mRNA transcripts processing in the region between exons 14 and 17 in wild-type animals (top) and myotonic pigs (bottom). Green bars represent the coding region sequence for the first CBS domain. The yellow line in exon 15 represents the Tyrosine residue participating in the formation of the selective chloride pore. Dashed lines represent RNA splicing processing. The red arrows represent the deletion breakpoints. The red lines and boxes represent the DNA deleted region. Note the absence of exons 15 and 16 in transcripts of myotonic animals, and the consequent lack of the tyrosine residue and part of the first CBS domain.

To gain better insight into the deletion identified in transcripts of myotonic pigs, we sequenced a relevant genomic region of 6,390 nucleotides including a region within intron 14 up to a region within intron 16 in five myotonic animals (A, B, C, D, and E) and in two non-myotonic animals (animals C1 and C2 of the control group). Sequencing analysis revealed a 4,165-nucleotide intragenic deletion in all tested myotonic animals (n = 5) (Fig. 2). The deletion comprised a partial fragment in the 3′ region of intron 14 (1,137 bp), exon 15 (214 bp), intron 15 (245 bp), exon 16 (134 bp), and a partial fragment in the 5′ region of intron 16 (2,435 bp). Sequencing analysis also mapped the exact breakpoint location at positions Chr18:6,912,538 and Chr18:6,916,702 (g. NC_010460.4 del 6912538_6916702).

Besides the large intragenic deletion, we identified 14 SNPs and a four-nucleotide insertion (g.23616_23617insATCG) in intron 14 and 21 SNP and a 60-nucleotide deletion (g.28130_28190del) in intron 16 in sequences of all myotonic animals (S2 Table). Those SNPs and that 60-nucleotide deletion were located away of exons/introns boundaries (more than 300 nucleotides), and no difference between the sequences of myotonic animals and reference sequence (NC_010460.4) was verified in these boundaries.

**Figure 3:**
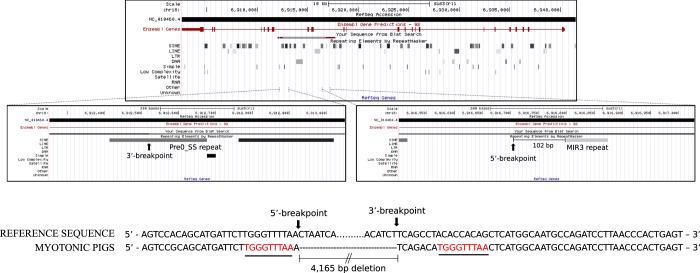
Nucleotide sequence analysis at the deletion junction of the *CLCN1* gene in myotonic pigs. Upper panel: Alignment of the genomic relevant consensus sequence of myotonic pigs against the pig genome. Note abundant short interspersed nuclear elements (SINE) within the *CLCN1* gene in pigs. Middle panels: Higher sequence resolution at both breakpoints indicates that the 5′-breakpoint is located 102 nucleotides downstream of a MIR3 repeat and the 3′-breakpoint lies within a Pre0_SS repetitive element. Lower panel: Analysis of the regions flanking both breakpoints reveal an identical TGGGTTTAA motif close to both breakpoints.

To investigate in more details the regions flanking both breakpoints, we aligned the genomic consensus sequence of myotonic pigs against an annotated pig genome (Sscrofa11.1/susScr11) using the Pig BLAT Search Genome tool of the UCSC Genome Browser. The RepeatMasker track analysis revealed abundant repeating sequences within the *CLCN1* gene, particularly short interspersed nuclear elements (SINE) in introns 14, 16, and 20. When zooming in at both breakpoints, we observed that the 5′-breakpoint in intron 14 was located 102 nucleotides downstream of a MIR3 repeat and the 3′-breakpoint in intron 16 lied within a Pre0_SS repeat. This analysis also revealed the presence of an identical 9-nucleotide sequence repeat flanking both breakpoints (Fig. 3).

### PCR-based genotyping assay

**Figure 4:**
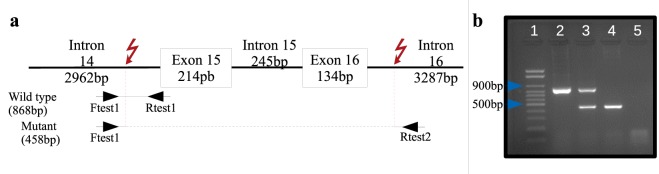
(**a**) Schematic representation of the region between introns 14 and 16. Black arrows indicate primers used in the PCR-based genotyping assay. Red arrows represent the boundaries of the deleted region. Amplicon sizes are indicated for both wild-type and mutant alleles. (**b**) Cropped picture of the 1.5% agarose gel electrophoresis showing typical results of the genotyping assay for Hereditary Myotonia in pigs. Well 1: Molecular-weight size marker. Well 2: Homozygous dominant (pig C1 of the control group) result (868 bp amplicon). Well 3: Heterozygous (pig K) result (868 bp and 458 bp amplicons). Well 4: Homozygous recessive (pig E) result (458 bp amplicon). Well 5: Negative control. Blue arrows indicate the DNA ladder’s fragment sizes 900 bp and 500 bp.

The PCR-based genotyping assay (Fig. 4a) optimized in the present study correctly identified the genotypes of all 22 pigs G22 group and the four unrelated non-myotonic animals (genotypes confirmed by sequencing) (Fig. 4b). All myotonic pigs were homozygous recessive for the 4,165-nucleotide deletion. The progenitors (pigs F, G, H, I, J, and K) of the affected animals and six (L, M, N, Q, R, and S) other animals of the family of myotonic pigs were considered heterozygous for the deletion. The remaining member (pig T) of the family of the myotonic pigs and all control animals were homozygous “wild type.” We also genotyped 150 unrelated and non-myotonic animals (farm group), and all of them were homozygous “wild type.”

### Expression of *CLCN1* gene transcripts

**Figure 5:**
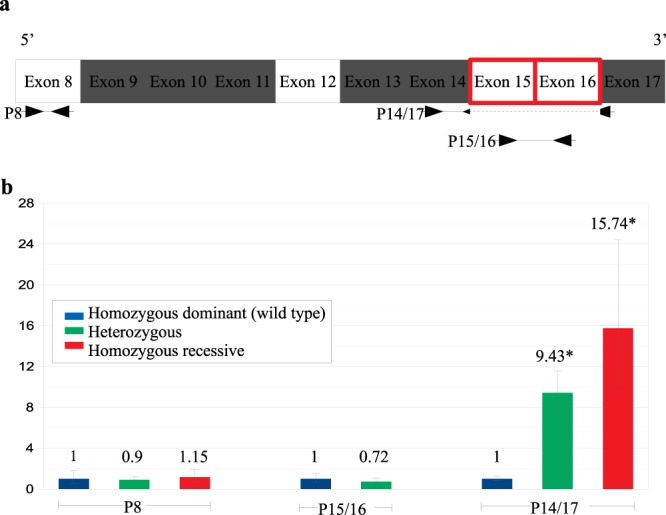
(**a**) Schematic representation of the positions of the real-time PCR amplicons along with the mRNA transcript. Rectangles with red-colored borders represent the deletion identified in myotonic pigs. Black arrows represent the annealing location of each primer. Note that the annealing location of the reverse primer used to generate the P14/17 amplicon is located at the exon 14 and 17 boundaries. (**b**) Relative gene expression of *CLCN1* gene transcripts among homozygous Wild Type, heterozygous, and homozygous recessive myotonic pigs. Bars represented the relative expression values with standard deviation of the means (S3 Table). The asterisk indicates statistical significance with p-value ≤ 0.05.

We sought to define the expression pattern of the *CLCN1* gene transcripts in homozygous recessive (animals O, P and V), heterozygous (animals H, K, and Q), and homozygous “wild type” pigs (controls animals 1, 2 and 3) (Fig. 5a). Amplification of a fragment of exon 8 (P8), upstream to the deleted region, indicated similar expression levels of the *CLCN1* gene among animals with all genotypes (S3 Table). As expected, amplification of a fragment of exons 15 and 16 (P15/16) at the deleted region was not detected in myotonic animals. Homozygous “wild type” and heterozygous non-myotonic pigs had similar levels of expression of the P15/16 fragment (S3 Table Fig. 5b). When we considered only the amplification of fragments spanning the exon 14 and exon 17 boundaries (P14/17) indicating the *CLCN1* transcript missing exons 15 and 16, homozygous recessive pigs expressed 1.67 and 15.73 times more the P14/17 alternative transcript than heterozygous and homozygous “wild type” animals. Moreover, mean expression levels of the P14/17 transcript in heterozygous animals was 9.42 times higher than that in homozygous “wild type” animals (S3 Table, Fig. 5b).

### *In silico* analysis of the CLC-1 protein

**Figure 6:**
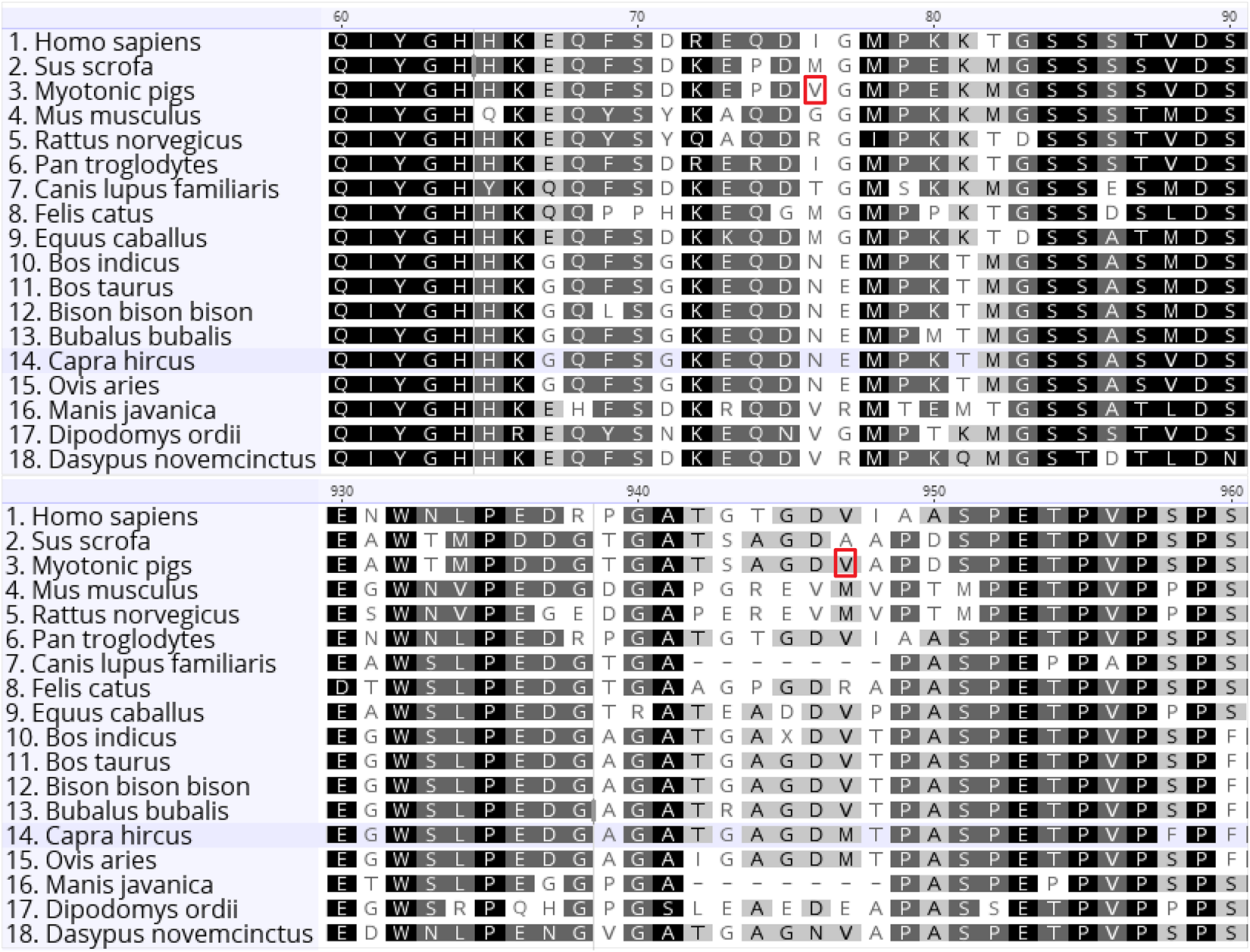
Alignment of the CLC-1 protein reference sequences of 16 mammalian species between positions 60–90 (upper panel) and 930–960 (lower panel). Access numbers of each reference sequence are provided in the supplementary data (S Table 5). Gray-scale backgrounds indicate conservation level of acid residues among the sequences; white < 60% similarity, light gray ≥ 60 and < 80% similarity, dark gray ≥ 80% and < 100% similarity, and black 100% similarity. Highlighted in red are the two residue substitutions identified in myotonic animals (p.Met76Val and p.Ala920Val).

The protein amino acid sequence in non-myotonic animals (control group) was identical than that in the reference sequence of the CLC-1 protein isoform X1 (XP_020934220.1), whereas the predicted protein sequence in myotonic animals (homozygous recessive) presented two substitutions (p.Met76Val and p.Ala920Val) and the absence of 116 amino acids (p.Gly528_Lys643). Alignment of the CLC-1 protein reference sequence in pigs and the reference sequences of 16 other mammalian species demonstrated that those methionine and alanine residues were not located within conserved regions (Fig. 6). That alignment also revealed an absolute identity of 57.8% and 92.2% among those species when considering the entire protein sequence or only the deleted polypeptide, respectively.

**Figure 7:**
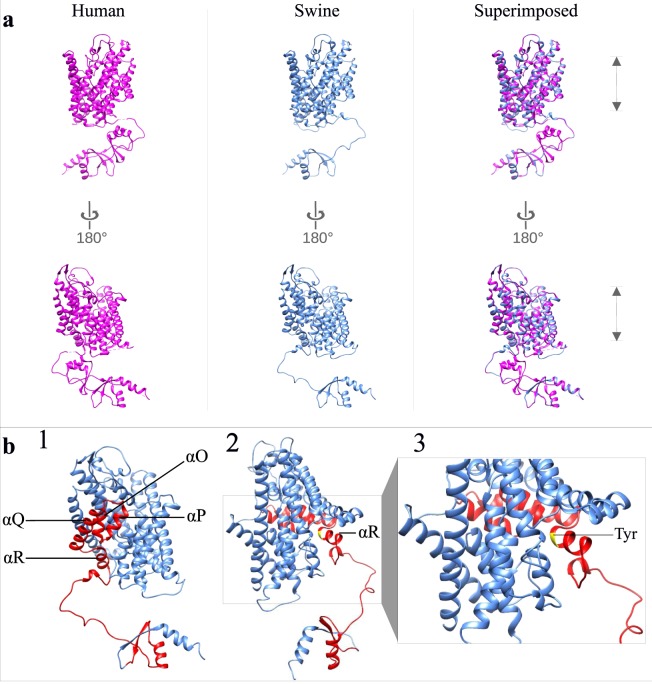
(**a**) Homology structure of the Chloride Channel protein 1 (Swine_CLC-1) in pigs. The tertiary structures of one subunit of the human (PDB – 6QVC - chain A) and swine CLC-1 homodimers are represented in magenta and cyan, respectively. The two subunits were also superimposed for comparison. Note the high similarity between the human model and the predicted structure in pigs. Gray arrows represent the membrane boundaries. (**b**) Tertiary structures of one subunit of CLC-1 homodimer in pigs. The consensus amino acids sequence between non-myotonic and myotonic animals is indicated in blue. The 116 amino acids deleted in myotonic animals are indicated in red. 1 - The deleted region in myotonic pigs spans from the C-terminal half of the α-helix O to the N-terminal half of the first CBS domain. 2 - The small yellow region within the α-helix R indicates the intracellular conserved tyrosine residue. 3 – Magnification of the tertiary structure indicated in 2.

**Figure 8:**
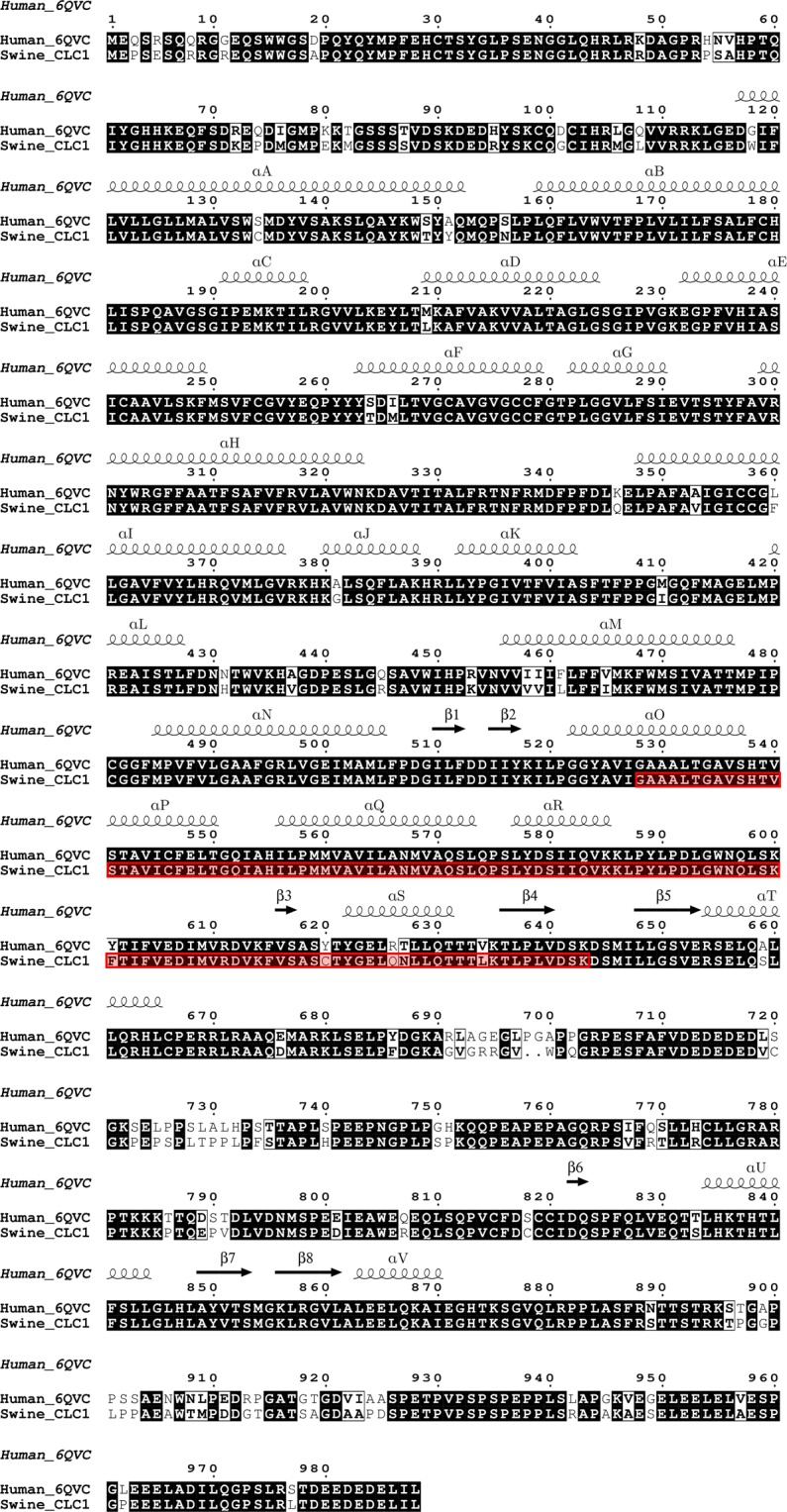
Alignment between the human CLC-1 protein sequence (6QVC) and the predicted protein sequence in pigs (Swine_CLC1). Secondary structures are indicated above the amino acid sequence. Alpha-helices were named according to the x-ray crystallographic structure proposed previously 30, conserved residues are shown in red. The red bars represent the absence of 116 amino acids in the deletion region of CLC-1 of myotonic pigs. The deleted region in myotonic pigs spans from the C-terminal half of the α-helix O to the N-terminal half of the first CBS domain.

We utilized three validated chloride channel structures (i.e., 1KPL, 3ORG, 6COY, and 6QVC)^27,30,34,35^ as models to predict the tertiary structure of the expected consensus protein sequence of non-myotonic pigs (control group). Considering the returning Z scores (data not shown), the best model for predicting the protein structure in pigs was the human CLC-1 chloride channel structure (i.e., 6QVC model). After model selection and optimization, the pig predicted protein structure was superimposed to the 6QVC structure (Fig. 7a). Protein sequences alignment revealed the identity of 95.04% (Fig. 8) between the predicted consensus sequence of non-myotonic pigs (control group) and the human protein structure (6QVC).

We used a similar homology-modeling approach in an attempt to predict the tertiary structure of the CLC-1 protein in myotonic pigs. However, none of the predicted structures satisfied the minimum global model quality values, and, therefore, we were not able to predict the tertiary structure in myotonic pigs. However, considering the tertiary structure predicted in non-myotonic animals, the deleted region corresponds to a protein region of 116 amino acids spanning from the C-terminal half of the α-helix O to the N-terminal half of the first CBS domain (Fig. 7b).

## Discussion

We describe here, for the first time, the clinical and molecular characterization of a form of Hereditary Myotonia in pigs. This disease was associated with a large genomic deletion in the *CLCN1* gene that had not been described in any animal species yet.

Similarly to clinical reports of Hereditary Myotonia affecting humans^7–13^ and other animals^16–22^, myotonic pigs exhibited muscle hypertrophy and stiffness, startle response, and warm-up phenomenon. Observed phenotypic variation was similar to that on buffalos^20^, cats^21^, goats^36^, and humans^9,10^ affected by mutations in the *CLCN1* gene. Percussion over the triceps produced the typical myotonic dimple described in cats^21^, goats^37^, and humans^9,24^ affected with Hereditary Myotonia. Myotonic pigs presented the classical pattern of myotonic discharges, as previously reported in humans and many other animals affected with Hereditary Myotonia^18–21,37–39^.

The major abnormality observed in the coding sequence of the *CLCN1* gene in all affected pigs was the absence of exons 15 and 16. Two transcripts of the pig *CLCN1* gene are available in the reference sequence database, variant X1 (XM_021078561.1) and variant X2 (XM_021078562.1). Alignment between the consensus coding sequence in pigs with that of variant X1 revealed the 348-nucleotide deletion in myotonic animals and high homology in the remaining sequence. Interestingly, exons 15 and 16 were also absent in variant X2 and, therefore, identical to the sequence identified in myotonic pigs. Exons 15 and 16 are also absent in transcripts of other four mammalian *CLCN1* genes, the *Bison bison* (XM_010834009.1), *Bos indicus* (XM_019959227.1), *Cercocebus atys* (XM_012057525.1), and *Macaca fascicularis* (XM_005551037.2). With the exception of *B. bison* coding sequence, all of those transcripts are considered alternative transcripts. The coding sequence of *B. bison* was predicted to encode a low-quality CLC-1 protein^40^. Thus, it is evident that transcripts without exons 15 and 16 are less frequent and usually are considered alternative transcripts.

Several findings in this study support the relevance of the detected deletion as the cause of Hereditary Myotonia in pigs. Myotonic pigs were all homozygous recessive for the deletion, whereas their progenitors were heterozygous, and all unrelated, non-myotonic pigs were homozygous “wild type.” A previous study investigating Hereditary Myotonia in cats associated the disease to the absence of exons 15 and 16 in the *CLCN1* gene due to a defective donor splicing site^21^. In horses^19^, the disease has been associated with single nucleotide polymorphisms (SNPs) in the same genomic region that was deleted in myotonic pigs. Moreover, at least six other distinct polymorphisms in the genomic region between introns 14 and 17 have been associated with Hereditary Myotonia in humans^41–44^. Those studies highlight the importance of exons 15 and 16 for the adequate function of CLC-1 channel.

The two missense mutations identified in this study (p.76 Met > Val and a p.920 Ala > Val) are unlikely the cause of Hereditary Myotonia in affected animals. The amino acids methionine and alanine are not inserted in conserved regions of the mammalian CLC-1 protein, and, more importantly, some species share them at the same positions (Fig. 6). Also, hydrophobic amino acids, such as methionine, alanine, and valine, can be replaced by amino acids of the same group without altering protein structure and impairing protein function^45,46^.

Sequence analysis of the genomic region between introns 14 and 17 (Fig. 2) revealed 14 nucleotide substitutions, one insertion, and two deletions. All of those changes except the large 4,165-nucleotide intragenic deletion were located more than 300 nucleotides away of exon-intron boundaries. Although it is not uncommon to associate human genetic disorders with intronic mutations^47,48^, including myotonic diseases such as myotonic dystrophy types 1 and 2^49,50^, we are not aware of studies linking a genomic deletion comprising exons to Hereditary Myotonia in any other species. This deletion likely does not have any epidemiological relevance to the swine industry because the myotonic pigs belonged to a small group of animals raised with no commercial purposes, and we did not observe the deletion in any of the 150 pigs used to validate the genotyping assay.

In this study, we sequenced the entire coding region (mRNA) of the *CLCN1* gene in four animals (two out of four non-myotonic pigs and two out of nine myotonic pigs). All myotonic animals had the mRNA coding region between exons 14 and 17 genotyped by direct sequencing, and the deletion was present in all animals. We also genotyped by direct sequencing the relevant genomic DNA sequence in five out of nine myotonic pigs. The fact that we did not perform those analyses in all myotonic pigs nor sequenced the entire *CLCN1* gene are limitations of this study. Moreover, all myotonic and non-myotonic animals included in the study had their genotype confirmed using the optimized PCR-based genotyping test. Another essential aspect of our study is that animal E (Fig. 2) was among the five pigs that we sequenced the genomic DNA region between intron 14 and intron 17. That pig had four myotonic siblings (A, B, C, and D) and was the progenitor (i.e., grandfather) of all four myotonic pigs in generation VI. We consider that this fact gives credibility to the findings described for pigs O, P, U, and V. It also suggests that the offspring of pig E may indeed present a similar genomic DNA deletion as their progenitor even though we did not sequence their DNA relevant region.

Transposable elements possess a vital role in facilitating evolutionary changes in genome^51^. However, repeating sequences are also potential sites for deleterious rearrangements causing genomic deletions and genetic disorders^52,53^. The frequency of gross genomic deletions is significantly higher within regions enriched with transposable elements^54^. Moreover, microhomology-mediated end joining (MMEJ), a known non-homologous recombination (NHR) mechanism, may also play a role in inducing large genomic deletions. MMEJ is an error-prone DNA double-strand break repair mechanism involving homologous pairing of 5–25 nucleotide sequences at broken ends^55^. In humans, large intragenic deletions are associated with both MMEJ events and deleterious rearrangements facilitated by transposable elements^56–58^. The effect of repeating sequences to genome stability and their consequences to health as well as the contribution of NHR events are much less studied in pigs. However, a recent study demonstrated that deletions in genes associated with reproductive traits in pigs contained Pre0_SS repeats^56^, suggesting that those repeating elements may be recombination hotspots. Another recent study identified NHR as the dominant deletion-forming mechanism in pigs^57^. The fact that the *CLCN1* gene in pig contained many Pre0_SS repeats within introns 14, 16, and 20 indicates the potential for the occurrence of genomic rearrangements in those regions. Indeed, the 3′-breakpoint lied within a Pre0_SS repeat. The breakpoint was also located seven nucleotides upstream to a TGGGTTTAA element that presented low homology with the reference sequence (Fig. 3). This 9-nucleotide element was identical to a sequence located one nucleotide upstream of the 5′-breakpoint. Therefore, we hypothesize that the developmental mechanism of the deletion identified in myotonic pigs may have likely involved an increased genomic instability of the Pre0_SS repeat at the 3′-breakpoint due to the accumulation of mutations producing TGGGTTTAA motif-containing fragments that led to non-homologous recombination events between both breakpoints.

Differences in the transcriptional levels of *CLCN1* have already been demonstrated between humans affected with dominant and recessive Hereditary Myotonia^59^. However, the present study compared the expression of *CLCN1* gene transcripts among homozygous “wild type,” heterozygous, and homozygous recessive individuals affected by the same causative mutation. We decided to optimize three distinct reactions to perform the gene expression analysis study. This was the case not because we intended to compare the expression of different transcripts among themselves but rather because we wanted to confirm the expression of those fragments among the three genotypes of pigs. Pigs of all three genotypes expressed similar levels of the *CLCN1* gene transcripts amplified with the P8 primer set. Homozygous “wild type” and the heterozygous animals expressed similar levels of the *CLCN1* gene transcripts amplified with the P15–16 primer set, and as expected, the homozygous recessive animals did not present expression values relative to this amplicon. Using the P14/17 primer set, we demonstrated that non-myotonic “wild type” animals expressed an alternative transcript of the *CLCN1* gene without exons 15 and 16. The annotated pig alternative transcript X2 (XM_021078562.1) is characterized by the absence of exons 15 and 16, and thus it is similar to the transcript resulting from the deletion. Although the transcript X2 was already available in the RefSeq database (NCBI), there was no previous experimental evidence demonstrating whether “wild-type” pigs express that alternate transcript, and this study provides such evidence. We were unable to find a previous study attributing function to an alternative transcript of the *CLCN1* gene. It is possible that transcripts of the variant X2 might encode an alternative isoform of the CLC-1 protein, including the formation of hybrid chloride channels in non-myotonic pigs. Hybrid CLC-1 channels resulting from the co-expression of distinct alternative transcripts have been associated with Hereditary Myotonia in humans^60,61^. It is possible that transcripts of the variant X2 might encode an alternative isoform of the CLC-1 protein, including the formation of hybrid chloride channels in non-myotonic pigs. In the present study, no protein expression assays were performed to determine the CLC-1 protein expression pattern among pigs of each genotype or the expression of another isoform of CLC-1. Although the absence of protein expression data may be a limitation in the understanding of Hereditary Myotonia in pigs, this analysis was beyond the scope of our work.

The CLC-1 protein is highly homologous among many organisms^62^. This characteristic was corroborated in the present study after the alignment of the predicted protein sequence in myotonic pigs with the pig reference sequence and sequences of other 16 mammals (S1 Figure). Modeling of the tertiary structure of transmembrane proteins is challenging because of the relatively low-sequence identity between the query and the templates^63^. However, transmembrane proteins share a similar fold pattern, even with homologies as low as 20%^64^. The tertiary structure predicted from the consensus protein sequence of non-myotonic control pigs presented 95.04% homology with the human reference sequence (Fig. 7)^35^. Despite the absence of any frameshift mutation, homology modeling of the CLC-1 tertiary structure of myotonic animals was not possible in this study because of the absence of the 116 amino acids resulting from the deletion. Repositioning of the amino acids upstream and downstream of the deleted region produced a poor-quality alignment with the reference sequence models, making it impossible to construct a satisfactory structure. Although it was not possible to model the cytoplasmic N-terminal region of the pig CLC-1 protein (Fig. 7), the CLC-1 protein predicted structure in pigs achieved the quality requirements to be considered a representative structure of pig CLC-1.

The intragenic deletion identified in this study contains part of the coding region encoding highly conserved domains of the CLC-1 protein. The helices H, I, P, and Q form part of the interface between the two monomers of CLC1. Furthermore, the helices G, H, I, P, and Q are important for CLC common gating process^65^. The absence of conserved domains responsible for the formation of the alpha-helices O, P, Q, and R in myotonic pigs probably impair the interaction between the two monomers and the function of common gate process^65^. Moreover, previous evidence revealed that the Myotonia-causing SNP A531V reduces whole-cell current density, protein expression levels, and shorter half-life^66^. The A531V missense mutation was proposed to cause a folding anomaly that prevents the protein from passing the quality control system in the endoplasmic reticulum^66^. The deletion identified in myotonic pigs includes the A531 amino acid; therefore, this deletion may have a negative effect on the biosynthesis and folding of CLC1 protein in pigs.

The α-helix R - deleted in myotonic pigs - is strategically located in the transition between the transmembrane and cytoplasmic domains. That α-helix motif includes a highly conserved tyrosine residue (Y578) that plays a crucial role in chloride ion-conducting pore formation^27,30,31,67^. The Cl^-^ permeation pathway is controlled at the intracellular side by this tyrosine residue^27,67,68^. Similarly to CLC-3 channels, the absence of this tyrosine residue probably alters anion selectivity and impairs the Cl^-^ permeation in CLC1 of myotonic pigs ^69^.

The genomic deletion led to the absence of 58.33% (35/60) of amino acids forming the first cytoplasmic CBS domain in myotonic pigs. Eukaryotic CLC-1 proteins carry a long cytoplasmic C-terminal region containing two CBS domains^30,31,34,35^. Although the function of those CBS domains in CLC-1 protein is not entirely well understood yet, previous studies proposed that those domains modulate channel activity by ATP binding and are influenced by pH and temperature^70,71^. The CBS domains make multiple contacts with the transmembrane helices αR and αD, which are composed of essential amino acids involved in Cl^-^ pore formation^30,35,68^. The CBS domains also interact with the loop connecting the transmembrane helices αH and αI; thus, CBS domains may influence the interactions between distinct alpha-helices of the transmembrane domain^30,35,68^. Therefore, it is likely that the partial absence of the first CBS domain of CLC1 in myotonic pigs leads to abnormalities in Cl^-^ pore formation and interaction between protein domains. The importance of the CBS domains to proper protein function is also highlighted by the fact that mutations involving those domains are often associated with Hereditary Myotonia with a similar inheritance pattern observed in humans^10,72,73^.

Previous studies investigating the relevance of specific regions of murine and human CLC-1 channels demonstrated that truncations within the region that was deleted in myotonic pigs impair both protein expression and chloride conductance^74,75^. For instance, failure of effective translation was observed in mutant clones producing truncations of the murine CLC-1 protein at positions 574 and 596. Those truncated CLC-1 proteins failed to generate appropriate chloride conductance patterns^73^. Moreover, another study demonstrated that truncation of the human CLC-1 channel at position 598 completely abolished chloride conductance^74^. Those studies evidence the importance of that region for the functionality of the chloride channel. Therefore, it is very likely that the absence of those 116 amino acids, between positions 528 and 643, in myotonic pigs impairs both protein expression and function of the CLC-1 chloride channel. Considering that the deleted mature mRNA was efficiently translated, and the amino acid sequences flanking the deleted region folded like native protein sequences, the CLC-1 protein in myotonic pigs would likely not be functional, and we would expect a reduction in chloride ion conductance of at least 70% since this is the minimum necessary for the occurrence of myotonia.

## Materials and Methods

### Animals and samples

In this study, we utilized 22 pigs (animals A to V –G22 group) that belonged to a family of 35 pigs. Out of those 22 pigs, nine were clinically affected by Hereditary Myotonia (animals A, B, C, D, E, O, P, U, and V). Four non-myotonic, unrelated, and clinically healthy large white pigs (C1, C2, C3, and C4 animals – control group) served as controls for all molecular studies. Additionally, 150 non-myotonic and unrelated large white pigs from six farms located in four different states in Brazil were utilized to validate the genotyping assay (animals 1 to 150 - farm group). Samples collection was performed using animals of the G22, control, and farm groups. Clinical evaluations were performed using G22 and control groups. Blood samples were obtained from all animals by auricular venipuncture using 21 G needles and stored in EDTA-containing Vacutainer blood collection tubes (Becton Dickinson). Muscular biopsy samples of the *gluteus medius* muscle were collected in all animals of the G22 and control groups. Each biopsy specimen was split into three smaller parts; one was frozen at −20 °C for DNA isolation, one was maintained in RNAlater (Ambion) and frozen at −20 °C for RNA isolation, and one was maintained in 10% formaldehyde solution for histological slides preparation.

### Use of experimental animals

All procedures were approved by the Institutional Animal Care and Use Committee (169/2015-CEUA) of São Paulo State University (UNESP) (protocol no. 169/2015). All methods were performed in accordance with the guidelines and regulations of CEUA.

### Clinical evaluation

Physical examination with emphasis on the musculoskeletal system was performed in all animals of the G22 and control groups. Pedigree records of the G22 group’s animals were obtained to construct the pedigree chart and characterize the inheritance pattern. Muscle biopsy sections of five affected animals and four control animals were stained with Hematoxylin and Eosin (H&E) and histologically evaluated.

Electromyography (EMG) was carried out on epaxial and proximal muscles (i.e., m. gluteus medius, m. deltoid, m. triceps brachii and m. longissimus dorsi) of five myotonic (animals A, B, C, D and E of G22 group) and two non-myotonic (animals C1 and C2 of control group) using concentric needle electrodes and a 2-channel Neuromax 1000 EMG portable system with the bandpass filter adjusted at 10 to 10,000 Hz.

### RT-PCR and sequence analysis of the complete cDNA sequence of the *CLCN1* gene

To characterize the full coding sequence of the pig *CLCN1* gene, we collected muscle biopsy samples of two myotonic animals (A and B of G22 group) and two non-myotonic animals (C1 and C2 of the control group). Total RNA was isolated from biopsy specimens using the RNeasy Mini Kit (Qiagen) and treated with RQ1 RNase-Free DNase (Promega). Relative purity, quality, and concentration of total RNA were determined by spectrometry (Nanodrop, 2000 Spectrophotometer, Thermo Scientific). The cDNA was synthesized using 800 ng of total RNA, random primers, and the enzyme ImProm-II Reverse Transcription System (Promega). Several primer sets were designed along the entire reference sequence (XM_021078561.1 [transcript variant X1]) (S4 Table). RT-PCR reactions were prepared to a final volume of 25 μL each, containing 2 μL of cDNA, 240 nM of each forward and reverse primer, 12.5 μL of the GoTaq Green Master Mix (Promega), and water nuclease-free q.s.p. Thermocycling conditions were optimized for each amplicon. All amplified products were purified and sequenced using RT-PCR primers and Sanger sequencing. Sequences were aligned against the reference coding sequences of the pig *CLCN1* gene (XM_021078561.1 [transcript variant X1] and XM_021078562.1 [transcript variant X2]) using the Geneious software version 10.2.3 (Biomatters).

### PCR amplification across the genomic deletion site and sequence analysis

Muscle biopsy samples of five myotonic animals (A, B, C, D, and E) and two non-myotonic animals (C1 and C2) were used to determine the sequence of the entire genomic region between introns 14 and 16 of the *CLCN1* gene. Several primer sets covering the target sequence were designed against the genomic reference sequence of the *CLCN1* gene (NC_010460.4) (S4 Table). Genomic DNA was isolated from muscle samples using the DNeasy Blood & Tissue kit (Qiagen). Relative purity, quality, and concentration of DNA were determined by spectrometry (Nanodrop, 2000 Spectrophotometer, Thermo Scientific). PCR reactions were set in a total of 25 μL, containing 2 μL of DNA, 0.4 μM of each forward and reverse primer, 12.5 μL of the GoTaq Green mix Master Mix (Promega), and water nuclease-free q.s.p. Amplified products were confirmed on agarose gel electrophoresis, purified, using the GenElute PCR Clean-Up Kit (Sigma-Aldrich), and sequenced using PCR primers and Sanger sequencing. Sequences were aligned against the reference genomic sequences of the pig *CLCN1* gene (NC_010460.4) and 16 mammalian reference sequences of the *CLCN1* gene (S6 Table) (alignment not shown). The alignments were made using the Geneious software version 10.2.3 (Biomatters).

### PCR-based genotyping assay

We used blood samples of three homozygous “wild type,” three heterozygous, and three homozygous recessive pigs (confirmed by sequencing of DNA region between introns 14 and 17) to develop and optimize a PCR-based genotyping assay. The two primer sets designed had a common forward primer in intron 14 and two distinct reverse primers, one in intron 14 and the other in intron 17. Genomic DNA was isolated using the ReliaPrep Blood gDNA Miniprep System kit (Promega), and relative purity, quality, and concentration of DNA were determined by spectrometry (Nanodrop, 2000 Spectrophotometer, Thermo Scientific). PCR reactions were set in a total of 25 μL, containing 2 μL of DNA (approximately 0.4 μg), 0.24 μM of the forward primer (Ftest1_5′TATGGCTTCCCCTGCATCTTT3′), 0.12 μM of each reverse primer (Rtest1_5′GTATGTGTAACTGACCCACT3′ and Rtest2_5′GGGTCCTTTGGGGGATGGA3′), 12.5 μL of the GoTaq Green Master Mix (Promega), and water-nuclease free q.s.p. *In silico* analysis was carried out to estimate PCR products size for each genotype; a single 868-nucleotide fragment for homozygous “wild type” animals, 868- and 458-nucleotide fragments for heterozygous animals, and a single 458-nucleotide fragment for homozygous recessive animals (Fig. 4). Amplified products were resolved on agarose gel electrophoresis to determine genotypes. The full-length agarose gel of the optimized PCR-based genotyping assay is available as supplementary material (S2, S3, and S4 Figures). The optimized genotyping assay was performed in all animals of the G22, control, and farm groups. PCR products of the animals of the G22 and control groups were sequenced to validate the genotyping assay results.

### Real-time PCR and expression analysis of *CLCN1* gene transcripts

To evaluate the expression of *CLCN1* mRNA transcripts, we used cDNA samples (muscle) of three homozygous “wild type,” three heterozygous, and three homozygous recessive pigs (confirmed by sequencing). Primer sets (S5 Table) were designed to amplify three different fragments of the *CLCN1* mRNA, a region of the exon 8 (P8) upstream to the deletion, a region between the exons 15 and 16 (P15/16) at the deletion, and a region between the exons 14 and 17 (P14/17) with the reverse primer at the splice junction (Fig. 5). A primer set was also designed to amplify a fragment of the pig *β-actin* mRNA (XM_021086047.1) that was used as the reference gene. Real-time PCR reactions were performed in triplicate in a total of 20 μL, containing 0.2 μM of each forward and reverse primer, 2 μL of cDNA (approximately 26 ng), 10 μL of GoTaq qPCR Master Mix (Applied Biosystems), and water nuclease-free q.s.p. A no-template control was included in each plate. All reactions were carried out using a 7500 Real-Time PCR System (Applied Biosystems, USA). PCR conditions were: initial denaturation at 95 °C for 10 min, 40 cycles at 95 °C for 15 sec and 60 °C for 60 sec, followed by a melting curve to confirm specific amplification of products. Relative gene expression of the *CLCN1* mRNA transcripts was performed using the comparative Ct method described elsewhere^76^.

### *In silico* protein analysis and modeling

The CLC-1 protein sequences of non-myotonic and myotonic pigs were predicted with the Geneious software version 10.2.3 using the full coding sequences of the *CLCN1* gene characterized before. The predicted protein sequences were compared to the protein reference sequences of the isoforms X1 (XP_020934220.1) and X2 (XP_020934221.1). The reference sequence of the pig isoform X1 was also aligned against reference sequences of 16 other mammalian species to identify conserved regions (S6 Table).

The tertiary structure of the CLC-1 protein was modeled using the consensus mRNA sequences of the non-myotonic and myotonic pigs using the protein structure homology-modeling server Swiss-Model^77^ and the 3D structural model of the CLC Chloride Channel PDB ID: 1KPL and 3ORG, and the 3D structural model of the human CLC-1 PDB ID: 6COY and 6QCV. Protein structure validation was performed using the ProSa-web tool^78^. The 3D model with the best overall quality, based on the calculated Z scores, was submitted to the UCSF Chimera Viewer tool for manual optimization and to obtain images. The program ESPript was used to perform alignment of protein sequences and secondary structure information from aligned sequences^79^.

### Author summary

Hereditary Myotonia is a genetic muscle disorder affecting the skeletal musculature of many animal species. In the present study, we described a large genomic deletion in the *CLCN1* gene associated with a recessive form of Hereditary Myotonia in pigs. This mutation results in the expression of a transcript with the exact same sequence of the alternative X2 transcript in pigs. Although the transcript in myotonic animals is identical compared to the alternative X2 transcript in non-affected pigs (the same 116 amino acids of the CLC-1 protein is missing in the X2 transcript of wild type and myotonic pigs), the transcript in the myotonic animals arose from an intragenic deletion. We also demonstrated that clinically normal pigs express low levels of the X2 alternative transcript, likely as the result of alternative splicing.

## Supplementary information

VIDEO 1

VIDEO 2

Supplementary Information

Dive Bomb Sound

## Data Availability

All data supporting the findings of this study are available within the article and its Supplementary Information Files or from the corresponding author on reasonable request.
