# Publisher Correction: A large intragenic deletion in the *CLCN1* gene causes Hereditary Myotonia in pigs

**DOI:** 10.1038/s41598-020-59863-x

**Published:** 2020-02-25

**Authors:** C. E. T. Araújo, C. M. C. Oliveira, J. D. Barbosa, J. P. Oliveira-Filho, L. A. L. Resende, P. R. Badial, J. P. Araujo-Junior, M. E. Mccue, A. S. Borges

**Affiliations:** 1 São Paulo State University (UNESP), School of Veterinary Medicine and Animal Science, Botucatu, São Paulo Brazil; 2 Instituto de Medicina Veterinária, Universidade Federal do Pará, Campus Castanhal, PA Brazil; 3 São Paulo State University (UNESP), Medical School, Botucatu, Brazil; 4 Department of Pathobiology and Population Medicine, College of Veterinary Medicine, Mississippi State University, Starkville, MS USA; 5 São Paulo State University (UNESP), Institute of Bioscience, Botucatu, Brazil; 6 College of Veterinary Medicine, University of Minnesota, St Paul, Minnesota 55108 USA

Correction to: *Scientific Reports* 10.1038/s41598-019-51286-7, published online 30 October 2019

A supplementary file containing a ‘dive bomb sound’ was omitted from the original version of this Article. This has been corrected in the HTML version of the Article; the PDF version was correct at time of publication.

