# Supplementary material for: A large intragenic deletion in the CLCN1 gene causes Hereditary Myotonia in pigs
Source: Sci Rep. 2019 Oct 30;9:15632. doi: 10.1038/s41598-019-51286-7 (PMC6821760; doi:10.1038/s41598-019-51286-7)
Supplement: Supplementary file 3 — Supplementary Information [file 41598_2019_51286_MOESM3_ESM.pdf]

A large intragenic deletion in the *CLCN1* gene causes Hereditary Myotonia in pigs

C. E. T. Araújo[1], Oliveira C. M. C.[2], Barbosa J. D.[2], J. P. Oliveira-Filho[1], Resende L. A. L.[3], Badial P. R.[4], Araujo-Junior J. P[5]., McCue M. E.[6], Borges A. S.[1*]

[1]São Paulo State University (UNESP), School of Veterinary Medicine and Animal Science, Botucatu, São Paulo, Brazil

[2]Instituto de Medicina Veterinária, Universidade Federal do Pará, *Campus* Castanhal-PA, Brazil

[3]São Paulo State University (UNESP), Medical School, Botucatu, Brazil

[4]Department of Pathobiology and Population Medicine, College of Veterinary Medicine, Mississippi State University, Starkville, MS, USA.

[5]São Paulo State University (UNESP), Institute for Biotechnology, Botucatu, Brazil

[6]College of Veterinary Medicine, University of Minnesota, St Paul, Minnesota 55108, USA

*Corresponding Author

Supplementary Information

S Table 1. Information about the family of 35 pigs including the G22 group (animals A to V).

Identification in manuscript	Identification in farm/university	Description	Phenotype	Genotype
1	1	Sow, SRD.	Normal	?
2	2	Boar, SRD.	Normal	?
3	3	First boar of the familly, SRD.	Normal	?
4	4	Sow, SRD.	Normal	?
5	5	Sow, SRD.	Normal	?
6	6	Boar, SRD.	Normal	?
7	7	Sow, SRD. (Mother of the first affected animals).	Normal	?
8	Large White	Sow, Large White, unrelated with the family.	Normal	?
A	Affected 1	Rearing pig, female	Myotonic (moderate)	del/del
B	Affected 2	Rearing pig, male	Myotonic (moderate)	del/del
C	Affected 3	Rearing pig, male	Myotonic (moderate)	del/del
D	Affected 4	Rearing pig, male	Myotonic (moderate)	del/del
E	Affected 5	Rearing pig, male, that became a boar and mate the large white female.	Myotonic (moderate)	del/del
F	1	Sow, ½ large white. Carrier 1.	Normal	N/del
G	4	Sow, ½ large white. Carrier 2.	Normal	N/del
H	6	Sow, ½ large white. Carrier 3.	Normal	N/del
I	130	Sow, ½ large white. Carrier 4.	Normal	N/del
J	3	Boar, ½ large white. Carrier 5.	Normal	N/del
K	200	Boar, ½ large white. Carrier 6.	Normal	N/del
L	2	Rearing pig, female.	Normal	N/del
M	11	Rearing pig, female.	Normal	N/del
N	30N	Rearing pig, female.	Normal	N/del
O	210	Rearing pig, female. Affected 6.	Myotonic (Mild)	del/del
P	230	Rearing pig, female. Affected 7.	Myotonic (Mild)	del/del
Q	20	Rearing pig, male.	Normal	N/del
R	21	Rearing pig, male.	Normal	N/del
S	33	Rearing pig, male.	Normal	N/del
T	13	Rearing pig, male.	Normal	N/N
U	30	Rearing pig, male.	Myotonic (intense)	del/del
V	34	Rearing pig, male.	Myotonic (intense)	del/del

S Table 2. Twenty-two single nucleotide polymorphisms (SNPs) and a four-nucleotide insertion (g.23616_23617insATCG) in intron 14 and 21 SNPs and a 60-nucleotide deletion (g.28130_28190del) in intron 16 of myotonic swine.

SNP	INTRON
g. 22638 A>G	14
g. 22663 T>G	14
g. 22688 A>G	14
g. 22713 A>G	14
g. 22737 T>C	14
g. 22781A>G	14
g. 22785 G>A	14
g. 22786 T>C	14
g. 22866 A>T	14
g. 22983 C>G	14
g. 22985 G>A	14
g. 23084 C>T	14
g. 23093 T>C	14
g. 23196 G>T	14
g. 23199 C>T	14
g. 23223 T>C	14
g. 23383 T>G	14
g. 23414 A>G	14
g. 23435 A>G	14
g. 23469 G>A	14
g. 23635 A>G	14
g. 23655 T>A	14
g. 27827 C>A	16
g. 27829 T>A	16
g. 27830 A>T	16
g. 27831 C>G	16
g. 27832 A>G	16
g. 27833 C>G	16
g. 27834 C>T	16
g. 27835 A>T	16
g. 27836 C>T	16
g. 27838 G>A	16
g. 27930 G>A	16
g. 27933 G>A	16
g. 27935 G>A	16
g. 28113 T>G	16
g. 28209 A>C	16
g. 28248 C>A	16
g. 28252 T>C	16
g. 28319 T>Y	16
g. 28320 C>M	16
g. 28322 G>A	16
g. 28336 G>A	16

S Table 3. ΔCT mean values of homozygous dominant, heterozygous, and homozygous recessive animals calculated for the P8, P15/16 and P14/17 real-time PCR products.

GENOTYPE	P8		P15/16		P14/17	
	ΔCT mean	ΔCT SD	ΔCT mean	ΔCT SD	ΔCT mean	ΔCT SD
Wild-type	9,526	0,847	8,455	0,703	15,814	0,286
Heterozygous	9,677	0,635	8,93	0,506	12,578	0,171
Homozygous (myotonic)	9,318	0,999	-	-	11,838	0,048

ΔCT SD the respective values of standard deviation for each genotype group. - were no aplification values for the product P15/16 for homozygous affected animals.

S Table 4. Primer sets designed to characterize the full coding sequence of the *CLCN1* gene in myotonic and normal pigs.

PRIMER ID		SEQUENCE 5'>3'	REGION
F1	*Forward*	TGACGGCGAGATGGCTATAA	UTR 5'
F2	*Forward*	GTACCAGTACATGCCCTTTG	Exon 1
F3	*Forward*	GCCATCACAAAGAGCAGT	Exon 2
F4	*Forward*	AGCTGGTGCATGGATTATG	Exon 3
F5	*Forward*	AAGGAATACCTCACCCTCAA	Exon 5
F6	*Forward*	TTGTCCACATTGCCAGCATC	Exon 6
F7	*Forward*	CTTGGAGGAGTGCTGTTTAG	Exons 7-8 boundary
F8	*Forward*	TGCCGTAAGGAACTACTGGC	Exon 8
F9	*Forward*	CTGGAATCGTGACCTTTGTC	Exon 11
F10	*Forward*	GGCTTCATGCCTGTGTTT	Exon 13
F11	*Forward*	GGTCAAGAAGCTACCCTACT	Exon 15
F12	*Forward*	TTCGCCTTTGTGGATGAG	Exon 17
F13	*Forward*	GCTCGTCCCACAAAGAAA	Exon 19
F14	*Forward*	GCCTCCACCTTGCTTATGT	Exon 22
R1	*Reverse*	CAAGTCCCATTCGGTGGATAC	Exon 3
R2	*Reverse*	AGCTGACCAGAGCCATTA	Exon 3
R3	*Reverse*	TGGTCTTCATTTCAGGGATTC	Exon 5
R4	*Reverse*	CGTCAGCATGTCAGTGTAATA	Exon 7
R5	*Reverse*	ATCCTTGTTCCACACGGCTA	Exon 8
R6	*Reverse*	CGGAAGTTGGTTCTGAAGAG	Exon 9
R7	*Reverse*	CCAGCCATGAATTGACCTAT	Exon 11
R8	*Reverse*	ATAGCCCCCAGGTAGGATC	Exon 14
R9	*Reverse*	CAAACTTCACATCACGTACCA	Exon 16
R10	*Reverse*	CAGACATCCTCATCCTCGT	Exon 17
R11	*Reverse*	GAACAGGGTGTGAGTCTTG	Exons 21-22 boundary
R12	*Reverse*	CCATCATCAGGCATGGTC	Exon 23
R13	*Reverse*	ATCAGTTCGTCCTCATCCT	Exon 23
R14	*Reverse*	CCCACTTTAGGAAATACCCTCTC	UTR 3'

S Table 5. Primers set used in real-time PCR reactions to amplify three different fragments of the *CLCN1* mRNA transcript, a region of the exon 8 (P8) upstream to the deletion, a region of exons 15 and 16 (P15/16) in the deleted region, and a region between the exons 14 and 17 (P14/17) downstream to the deletion with the reverse primer located at the exons 14 and 17 boundary, and Beta actin housekeeping gene.

Amplicon	Amplicon size (pb)	Primer sequence	Type
P8	85	TGCCGTAAGGAACTACTGGC	*Forward*
		ATCCTTGTTCCACACGGCTA	*Reverse*
P15/16	98	GGTCAAGAAGCTACCCTACT	*Forward*
		CAAACTTCACATCACGTACCA	*Reverse*
P14/17	84	CTTCCCGGACGGCATTTTA	*Forward*
		AGCAGGATCATGGAATCAATG	*Reverse*
Bact	86	CATTGTCCACCTTCCAGCAGATGT	*Forward*
		CTAGAAGCATTTGCGGTGGACGAT	*Reverse*

S Table 6. Reference sequences of the chloride channel protein 1 (CLC1) of 15 mammalian species aligned with the predicted CLC1 isoform X1 in pigs.

Sequence ID	Acess number<br>Refseq - NCBI	Species	Protein isoform
1	NP_000074	Homo sapiens	X1
2	XP_020934220	Sus scrofa	X1
3	NP_001291956.1	Felis catus	-
4	NP_001003124.1	Canis lupus familiaris	-
5	NP_001277845.1	Bubalus bubalis	-
6	NP_038519.1	Mus musculus	-
7	NP_037279.1	Rattus norvegicus	-
8	XP_019814784.1	Bos indicus	X1
9	XP_016813811.1	Pan troglodytes	X1
10	XP_013819008.1	Capra hircus	X1
11	XP_004008185.3	Ovies aries	X1
12	XP_001915671.2	Equus caballus	-
13	XP_017527433.1	Manis javanica	-
14	XP_012877091.1	Dipodomys ordii	-
15	XP_004474189.1	Dasypus novemcinctus	-

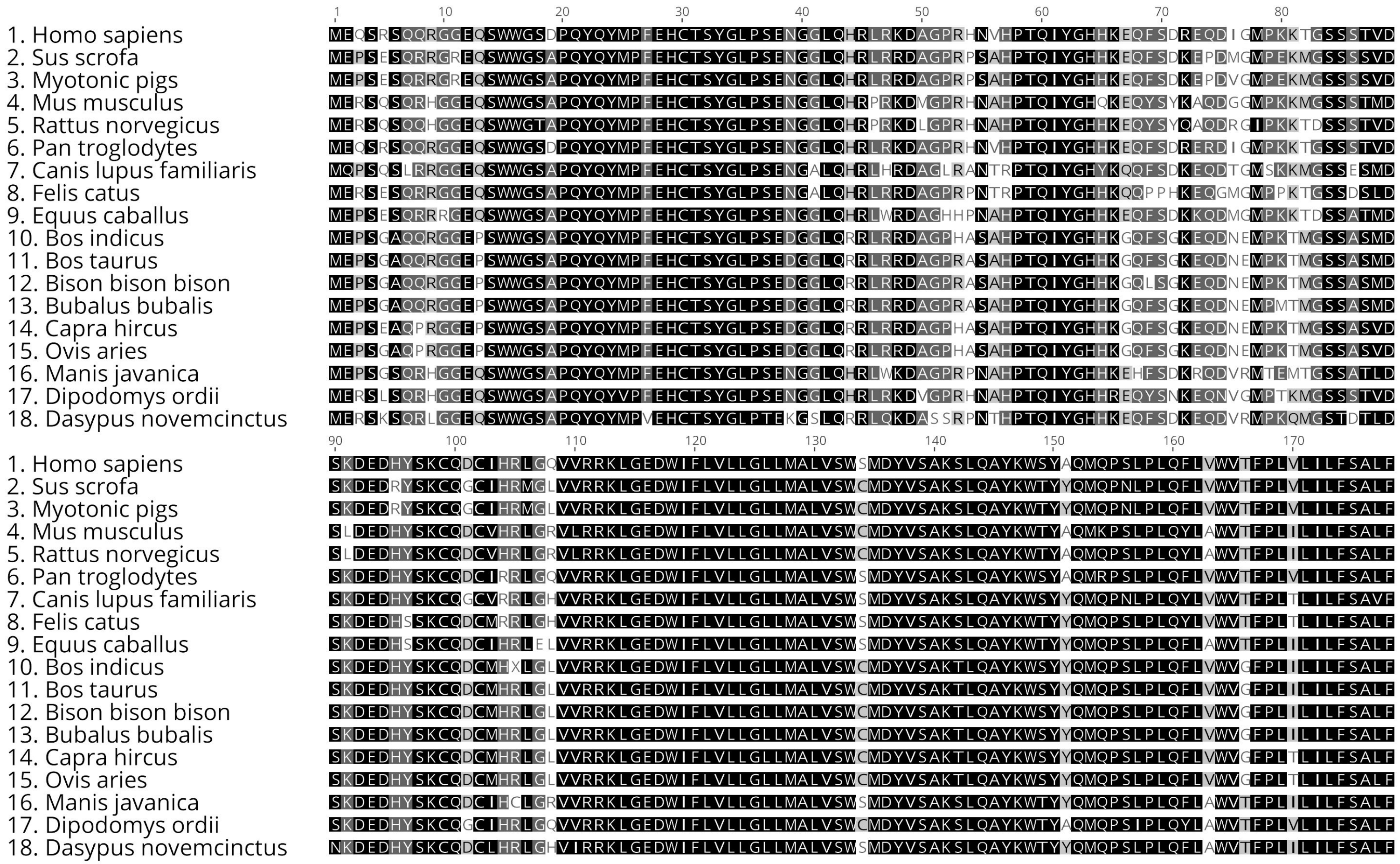
1
10
20
30
40
50
60
70
80
1. Homo sapiens
2. Sus scrofa
3. Myotonic pigs
4. Mus musculus
5. Rattus norvegicus
6. Pan troglodytes
7. Canis lupus familiaris
8. Felis catus
9. Equus caballus
10. Bos indicus
11. Bos taurus
12. Bison bison bison
13. Bubalus bubalis
14. Capra hircus
15. Ovis aries
16. Manis javanica
17. Dipodomys ordii
18. Dasypus novemcinctus
90
100
110
120
130
140
150
160
170
1. Homo sapiens
2. Sus scrofa
3. Myotonic pigs
4. Mus musculus
5. Rattus norvegicus
6. Pan troglodytes
7. Canis lupus familiaris
8. Felis catus
9. Equus caballus
10. Bos indicus
11. Bos taurus
12. Bison bison bison
13. Bubalus bubalis
14. Capra hircus
15. Ovis aries
16. Manis javanica
17. Dipodomys ordii
18. Dasypus novemcinctus

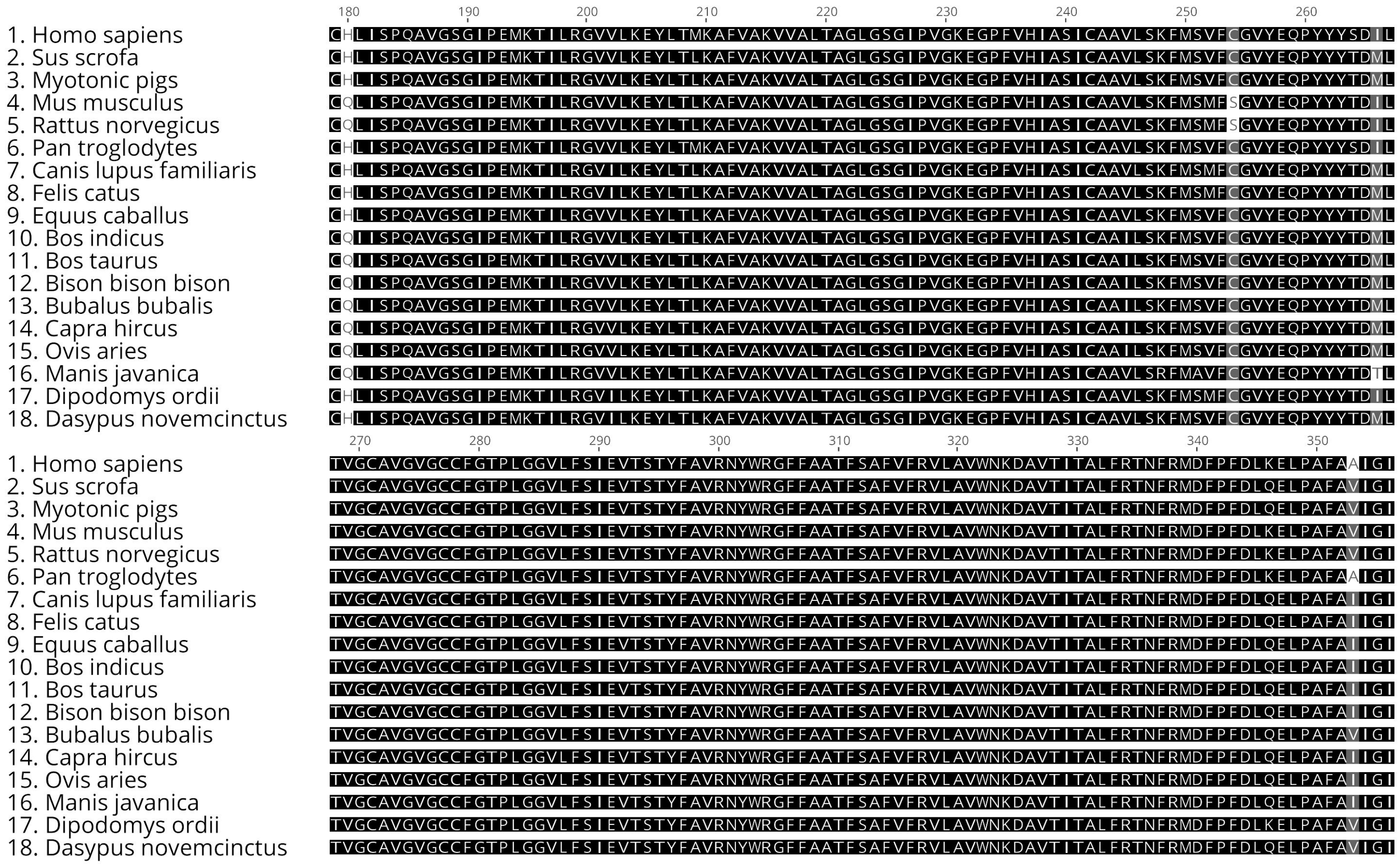
180 190 200 210 220 230 240 250 260
1. Homo sapiens
2. Sus scrofa
3. Myotonic pigs
4. Mus musculus
5. Rattus norvegicus
6. Pan troglodytes
7. Canis lupus familiaris
8. Felis catus
9. Equus caballus
10. Bos indicus
11. Bos taurus
12. Bison bison bison
13. Bubalus bubalis
14. Capra hircus
15. Ovis aries
16. Manis javanica
17. Dipodomys ordii
18. Dasypus novemcinctus
270 280 290 300 310 320 330 340 350

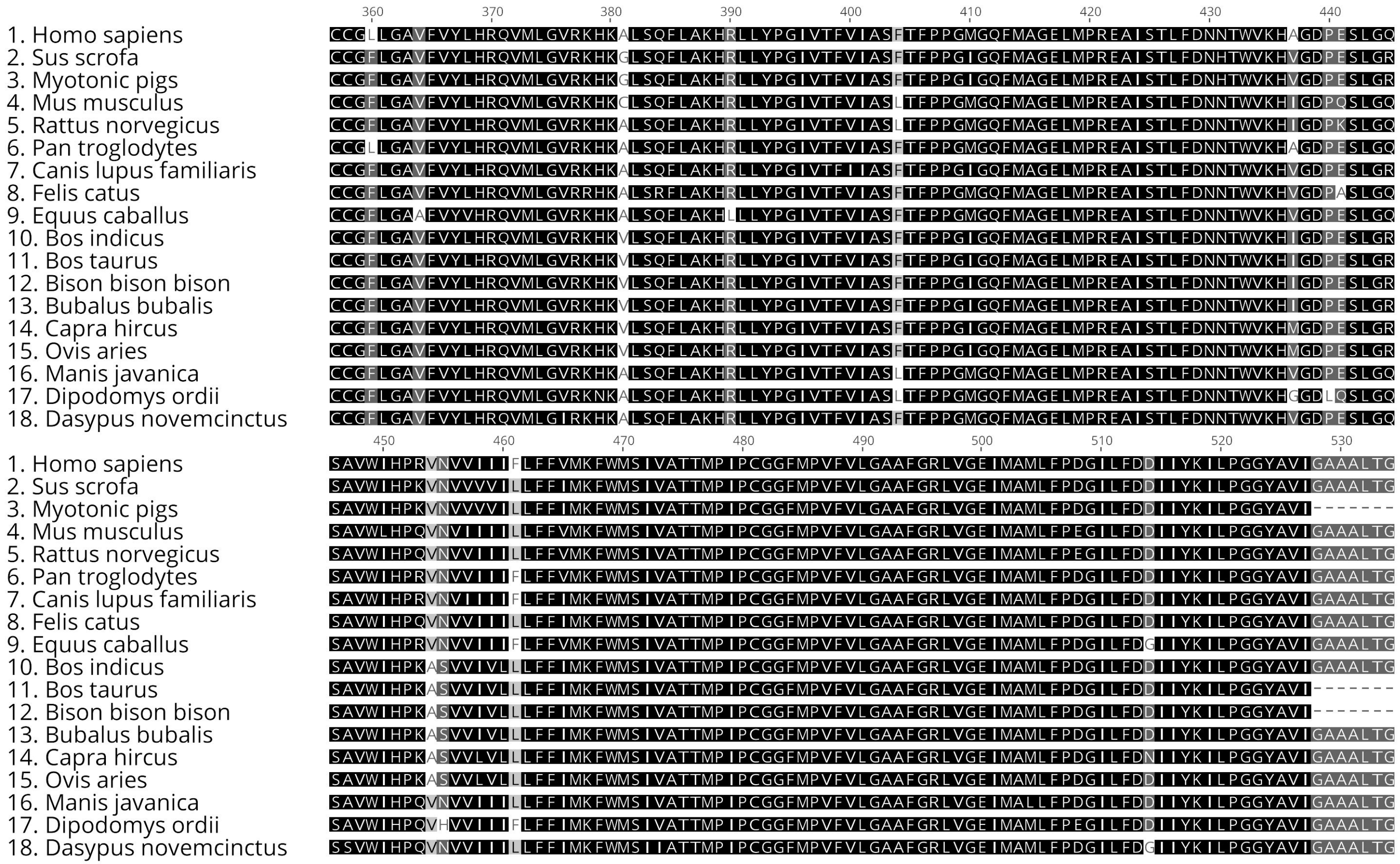
360 370 380 390 400 410 420 430 440
1. Homo sapiens
2. Sus scrofa
3. Myotonic pigs
4. Mus musculus
5. Rattus norvegicus
6. Pan troglodytes
7. Canis lupus familiaris
8. Felis catus
9. Equus caballus
10. Bos indicus
11. Bos taurus
12. Bison bison bison
13. Bubalus bubalis
14. Capra hircus
15. Ovis aries
16. Manis javanica
17. Dipodomys ordii
18. Dasypus novemcinctus
450 460 470 480 490 500 510 520 530

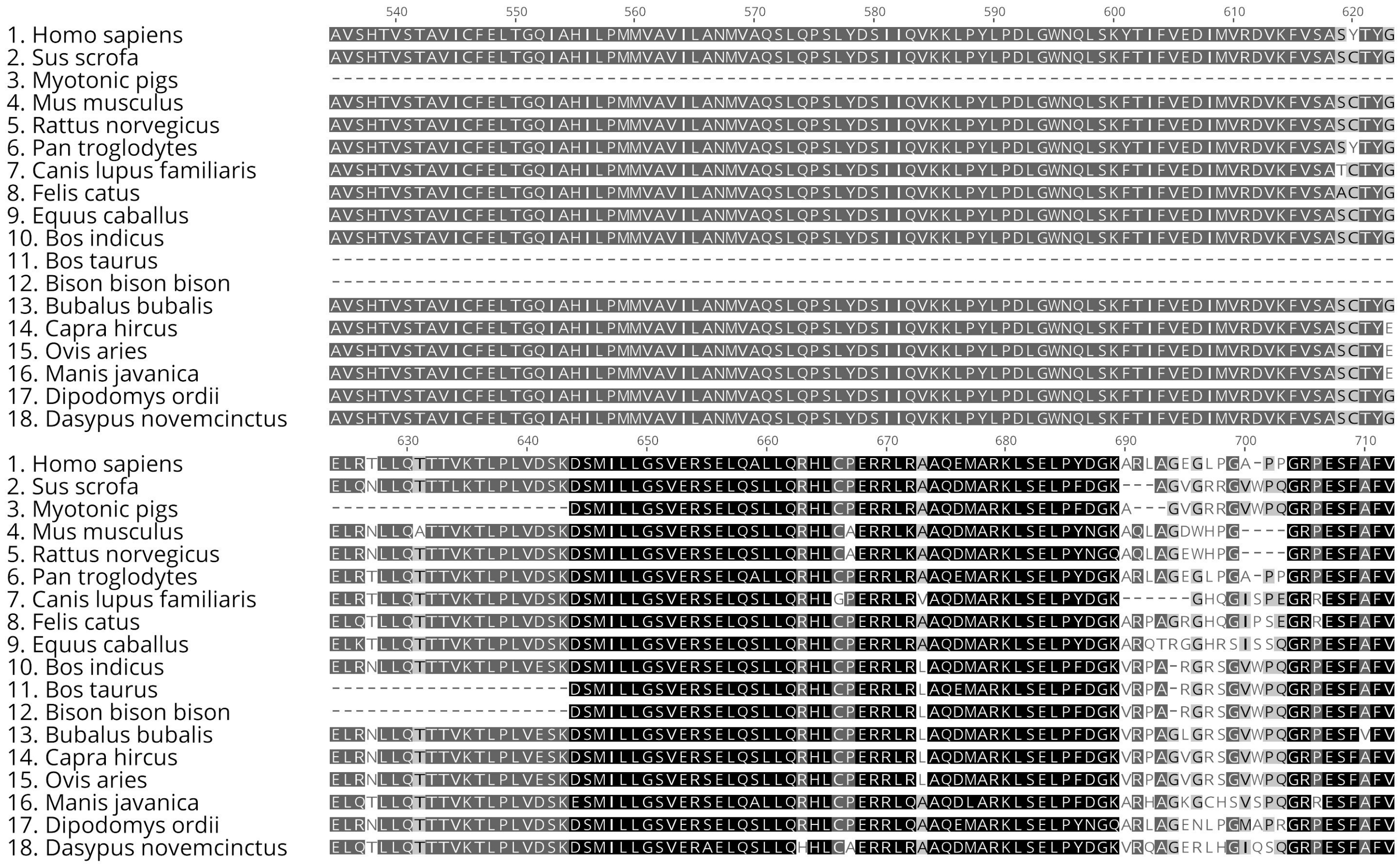

540 550 560 570 580 590 600 610 620
1. Homo sapiens AVSHTVSTAVICFELTGQIAHILPMMVAVILANMVAQSLQPSLYDSIIQVKKLPYLPDLGWNQLSKYTIFVEDIMVRDVKFVSASYTYG
2. Sus scrofa AVSHTVSTAVICFELTGQIAHILPMMVAVILANMVAQSLQPSLYDSIIQVKKLPYLPDLGWNQLSKFTIFVEDIMVRDVKFVSASCTYG
3. Myotonic pigs ---
4. Mus musculus AVSHTVSTAVICFELTGQIAHILPMMVAVILANMVAQSLQPSLYDSIIQVKKLPYLPDLGWNQLSKFTIFVEDIMVRDVKFVSASCTYG
5. Rattus norvegicus AVSHTVSTAVICFELTGQIAHILPMMVAVILANMVAQSLQPSLYDSIIQVKKLPYLPDLGWNQLSKFTIFVEDIMVRDVKFVSASCTYG
6. Pan troglodytes AVSHTVSTAVICFELTGQIAHILPMMVAVILANMVAQSLQPSLYDSIIQVKKLPYLPDLGWNQLSKYTIFVEDIMVRDVKFVSASYTYG
7. Canis lupus familiaris AVSHTVSTAVICFELTGQIAHILPMMVAVILANMVAQSLQPSLYDSIIQVKKLPYLPDLGWNQLSKFTIFVEDIMVRDVKFVSATCTYG
8. Felis catus AVSHTVSTAVICFELTGQIAHILPMMVAVILANMVAQSLQPSLYDSIIQVKKLPYLPDLGWNQLSKFTIFVEDIMVRDVKFVSAACTYG
9. Equus caballus AVSHTVSTAVICFELTGQIAHILPMMVAVILANMVAQSLQPSLYDSIIQVKKLPYLPDLGWNQLSKFTIFVEDIMVRDVKFVSASCTYG
10. Bos indicus AVSHTVSTAVICFELTGQIAHILPMMVAVILANMVAQSLQPSLYDSIIQVKKLPYLPDLGWNQLSKFTIFVEDIMVRDVKFVSASCTYG
11. Bos taurus ---
12. Bison bison bison ---
13. Bubalus bubalis AVSHTVSTAVICFELTGQIAHILPMMVAVILANMVAQSLQPSLYDSIIQVKKLPYLPDLGWNQLSKFTIFVEDIMVRDVKFVSASCTYG
14. Capra hircus AVSHTVSTAVICFELTGQIAHILPMMVAVILANMVAQSLQPSLYDSIIQVKKLPYLPDLGWNQLSKFTIFVEDIMVRDVKFVSASCTYE
15. Ovis aries AVSHTVSTAVICFELTGQIAHILPMMVAVILANMVAQSLQPSLYDSIIQVKKLPYLPDLGWNQLSKFTIFVEDIMVRDVKFVSASCTYE
16. Manis javanica AVSHTVSTAVICFELTGQIAHILPMMVAVILANMVAQSLQPSLYDSIIQVKKLPYLPDLGWNQLSKFTIFVEDIMVRDVKFVSASCTYE
17. Dipodomys ordii AVSHTVSTAVICFELTGQIAHILPMMVAVILANMVAQSLQPSLYDSIIQVKKLPYLPDLGWNQLSKFTIFVEDIMVRDVKFVSASCTYG
18. Dasypus novemcinctus AVSHTVSTAVICFELTGQIAHILPMMVAVILANMVAQSLQPSLYDSIIQVKKLPYLPDLGWNQLSKFTIFVEDIMVRDVKFVSASCTYG
630 640 650 660 670 680 690 700 710
1. Homo sapiens ELRTLLQTTTVKTLPLVDSKDSMILLGSVERSELQALLQRHLCPERRLRAAQEMARKLSELPYDGKARLAGEGLPGA-PPGRPESFAFV
2. Sus scrofa ELQNLLQTTTLKTLPLVDSKDSMILLGSVERSELQSLLQRHLCPERRLRAAQDMARKLSELPFDGK---AGVGRRGVWPQGRPESFAFV
3. Myotonic pigs --------------------DSMILLGSVERSELQSLLQRHLCPERRLRAAQDMARKLSELPFDGKA---GVGRRGVWPQGRPESFAFV
4. Mus musculus ELRNLLQATTVKTLPLVDSKDSMILLGSVERSELQSLLQRHLCAERRLKAAQDMARKLSELPYNGKAQLAGDWHPG----GRPESFAFV
5. Rattus norvegicus ELRNLLQTTTVKTLPLVDSKDSMILLGSVERSELQSLLQRHLCAERRLKAAQDMARKLSELPYNGQAQLAGEWHPG----GRPESFAFV
6. Pan troglodytes ELRTLLQTTTVKTLPLVDSKDSMILLGSVERSELQALLQRHLCPERRLRAAQEMARKLSELPYDGKARLAGEGLPGA-PPGRPESFAFV
7. Canis lupus familiaris ELRTLLQTTTVKTLPLVDSKDSMILLGSVERSELQSLLQRHLGPERRLRVAQDMARKLSELPYDGK------GHQGISPEGRRESFAFV
8. Felis catus ELQTLLQTTTVKTLPLVDSKDSMILLGSVERSELQSLLQRHLCPERRLRAAQDMARKLSELPYDGKARPAGRGHQGIPSEGRRESFAFV
9. Equus caballus ELKTLLQTTTVKTLPLVDSKDSMILLGSVERSELQSLLQRHLCPERRLRAAQDMARKLSELPYDGKARQTRGGHRSISSQGRPESFAFV
10. Bos indicus ELRNLLQTTTVKTLPLVESKDSMILLGSVERSELQSLLQRHLCPERRLRLAQDMARKLSELPFDGKVRPA-RGRSGVWPQGRPESFAFV
11. Bos taurus --------------------DSMILLGSVERSELQSLLQRHLCPERRLRLAQEMARKLSELPFDGKVRPA-RGRSGVWPQGRPESFAFV
12. Bison bison bison --------------------DSMILLGSVERSELQSLLQRHLCPERRLRLAQDMARKLSELPFDGKVRPA-RGRSGVWPQGRPESFAFV
13. Bubalus bubalis ELRNLLQTTTVKTLPLVESKDSMILLGSVERSELQSLLQRHLCPERRLRLAQDMARKLSELPFDGKVRPAGLGRSGVWPQGRPESFVFV
14. Capra hircus ELRNLLQTTTVKTLPLVESKDSMILLGSVERSELQSLLQRHLCPERRLRLAQDMARKLSELPFDGKVRPAGVGRSGVWPQGRPESFAFV
15. Ovis aries ELRNLLQTTTVKTLPLVESKDSMILLGSVERSELQSLLQRHLCPERRLRLAQDMARKLSELPFDGKVRPAGVGRSGVWPQGRPESFAFV
16. Manis javanica ELQTLLQTTTVKTLPLVDSKESMILLGSVERSELQALLQRHLCPERRLQAAQDLARKLSELPFDGKARHAGKGCHSVSPQGRRESFAFV
17. Dipodomys ordii ELRNLLQTTTVKTLPLVDSKDSMILLGSVERSELQSLLQRHLCPERRLQAAQEMARKLSELPYNGQARLAGENLPGMAPRGRPESFAFV
18. Dasypus novemcinctus ELQTLLQTTTVKTLPLVDSKDSMILLGSVERAELQSLLQHHLCAERRLRAAQDMARKLSELPYDGKVRQAGERLHGIQSQGRPESFAFV

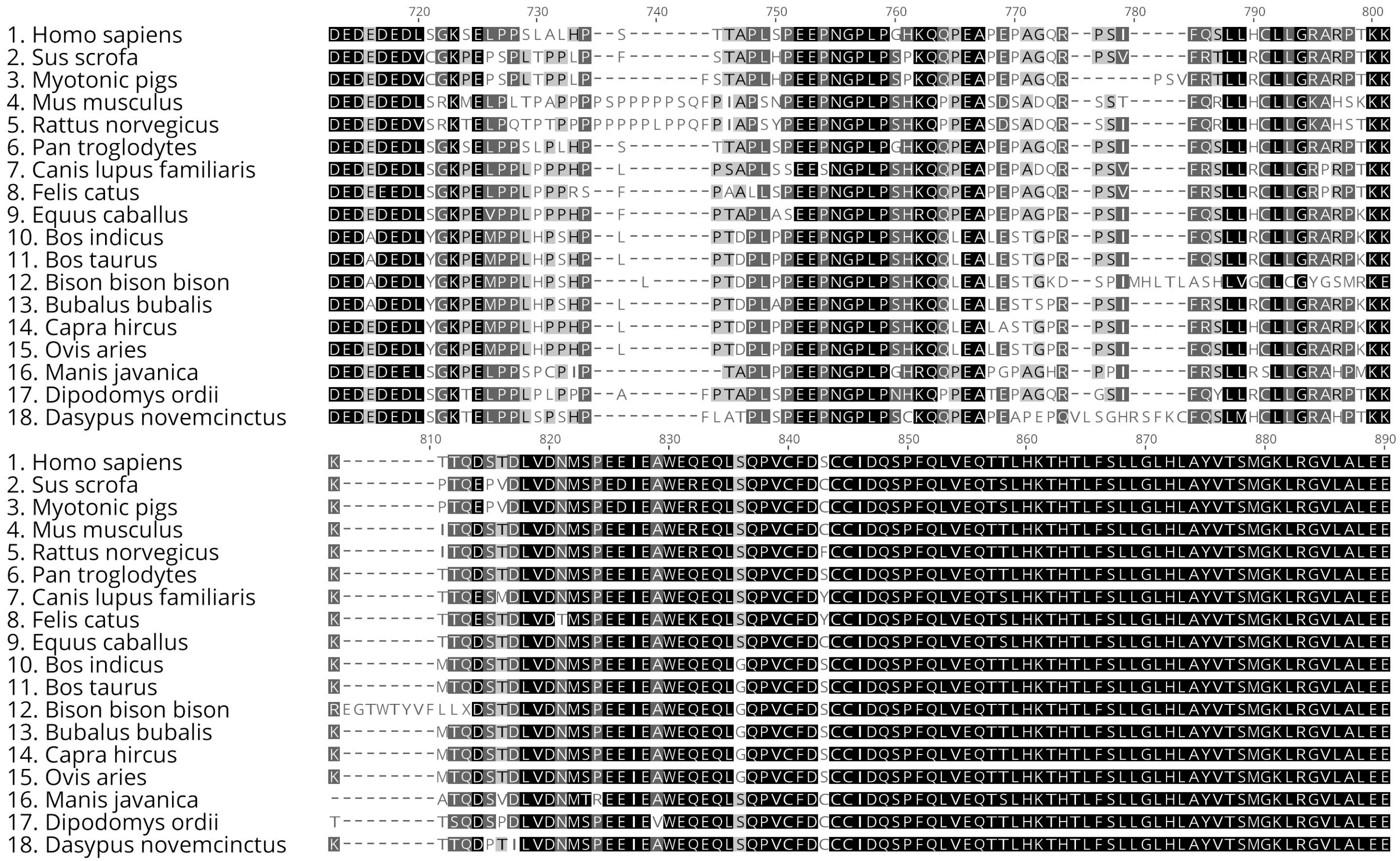
720 730 740 750 760 770 780 790 800
1. Homo sapiens
2. Sus scrofa
3. Myotonic pigs
4. Mus musculus
5. Rattus norvegicus
6. Pan troglodytes
7. Canis lupus familiaris
8. Felis catus
9. Equus caballus
10. Bos indicus
11. Bos taurus
12. Bison bison bison
13. Bubalus bubalis
14. Capra hircus
15. Ovis aries
16. Manis javanica
17. Dipodomys ordii
18. Dasypus novemcinctus
810 820 830 840 850 860 870 880 890
1. Homo sapiens
2. Sus scrofa
3. Myotonic pigs
4. Mus musculus
5. Rattus norvegicus
6. Pan troglodytes
7. Canis lupus familiaris
8. Felis catus
9. Equus caballus
10. Bos indicus
11. Bos taurus
12. Bison bison bison
13. Bubalus bubalis
14. Capra hircus
15. Ovis aries
16. Manis javanica
17. Dipodomys ordii
18. Dasypus novemcinctus

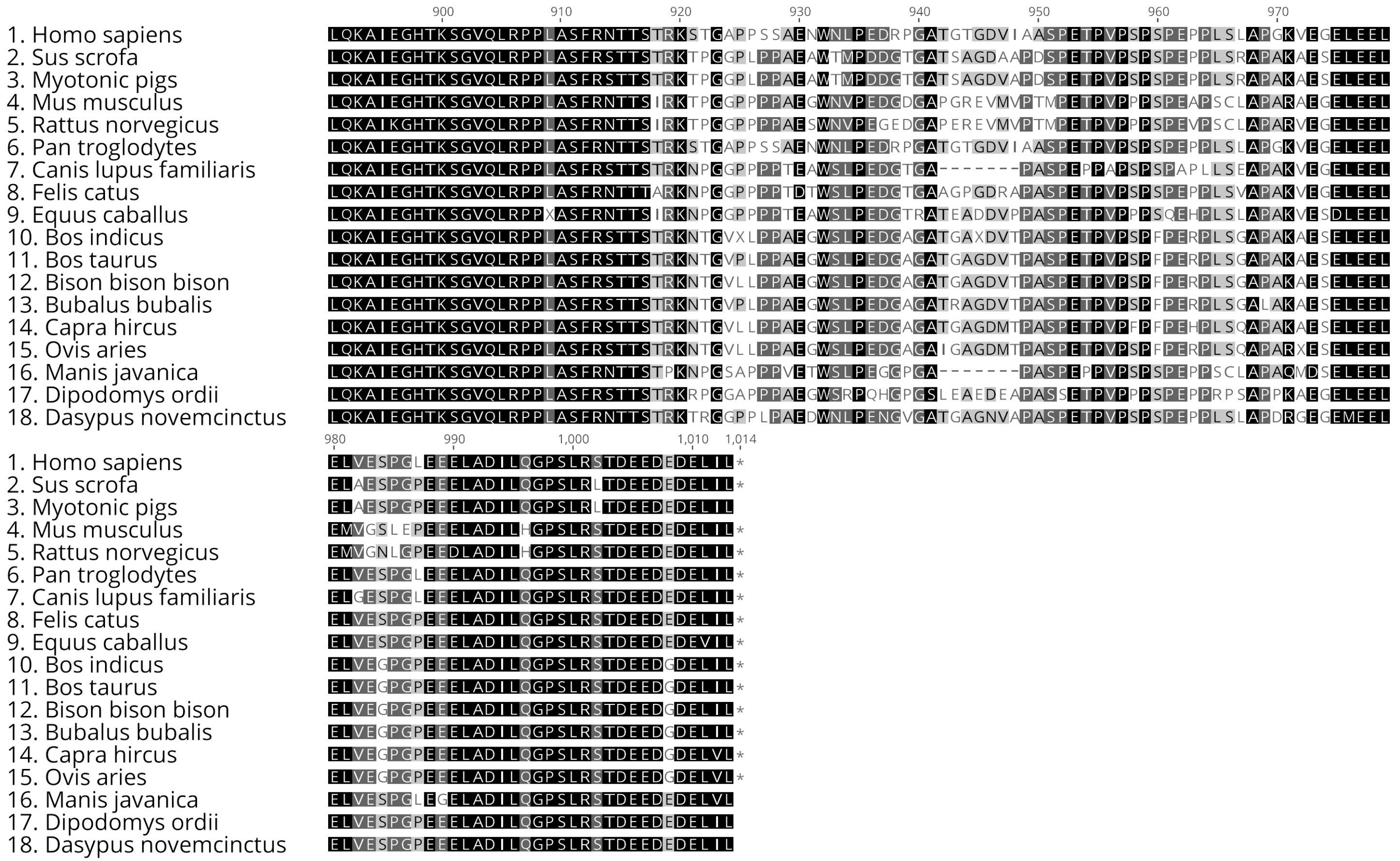

S1 Figure. Alignment of the predicted Chloride Channel protein 1 (CLC1) sequence in pigs and the CLC1 protein sequences of other 16 mammals.

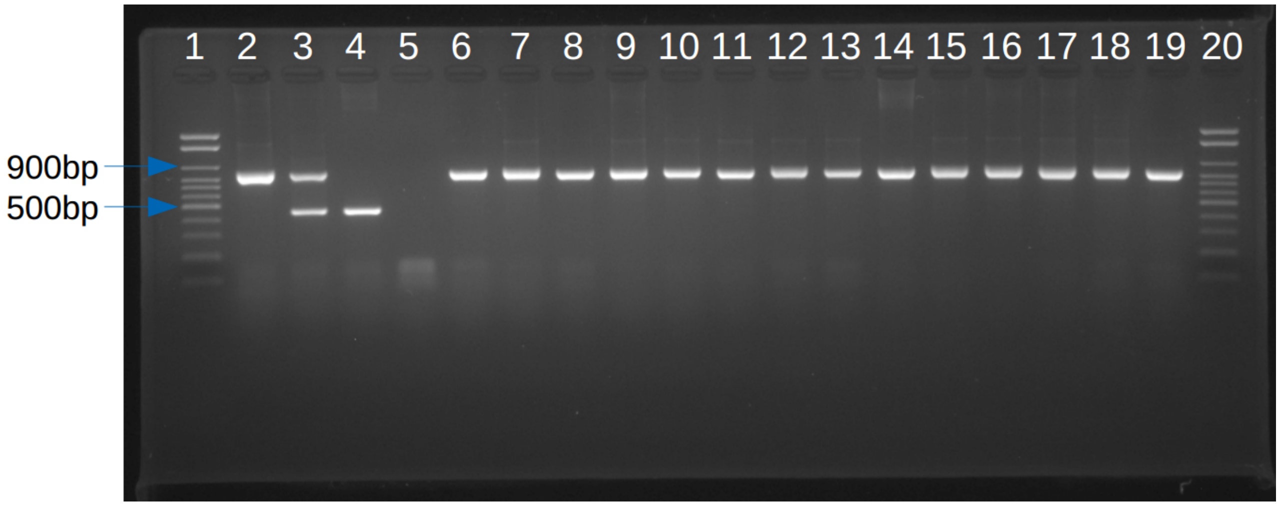

S2 Figure. Full-length 1.5% agarose gel electrophoresis showing typical results of the genotyping assay for Hereditary Myotonia in pigs. Well 1: Molecular-weight size marker. Well 2: Homozygous dominant (pig C1 of the control group) result (868 bp amplicon). Well 3: Heterozygous (pig K) result (868 bp and 458 bp amplicons). Well 4: Homozygous recessive (pig E) result (458 bp amplicon). Well 5: Negative control. Well 6 to 19: Homozygous dominant results (pigs 1 to 14 of the farm group; non-myotonic and unrelated large white pigs). Blue arrows indicate the DNA ladder's fragment sizes 900 bp and 500 bp.

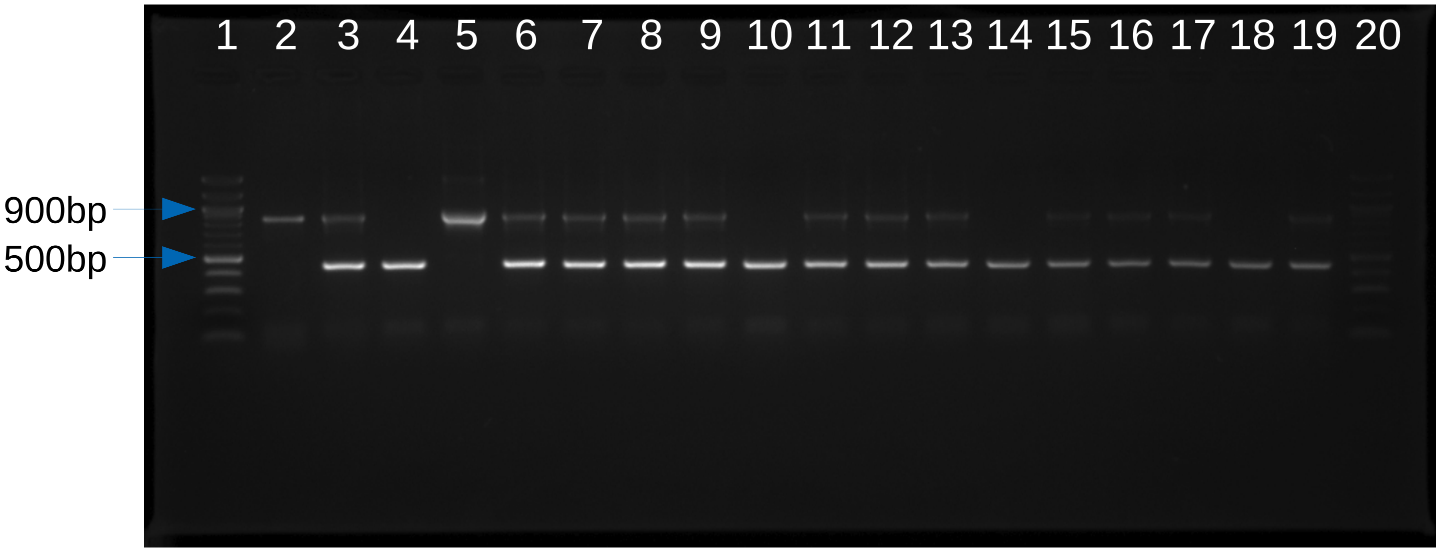

S3 Figure. Full-length 1.5% agarose gel electrophoresis showing representative results of the genotyping assay for Hereditary Myotonia in pigs. Wells 1 and 20: Molecular-weight size marker. Well 2: Homozygous dominant control (868 bp amplicon – animal C1 of control group). Well 3: Heterozygous control result (868 bp and 458 bp amplicons - animal F of G22 group). Well 4: Homozygous recessive result (458 bp amplicon - animal U of G22 group). Homozygou wild type result in well 5: animal T of G22 group . Heterozygous results in wells 6, 7, 8, 9, 11, 12, 13, 15, 16, 17 and 19 respectively for animals L, J, G, H, M, Q, R, N, S, I, and K (G22 group). Homozygous recessive results in wells 10, 14, and 18 respectively for animals V, O, and P (G22 group). Blue arrows indicate the DNA ladder's fragment sizes 900 bp and 500 bp.

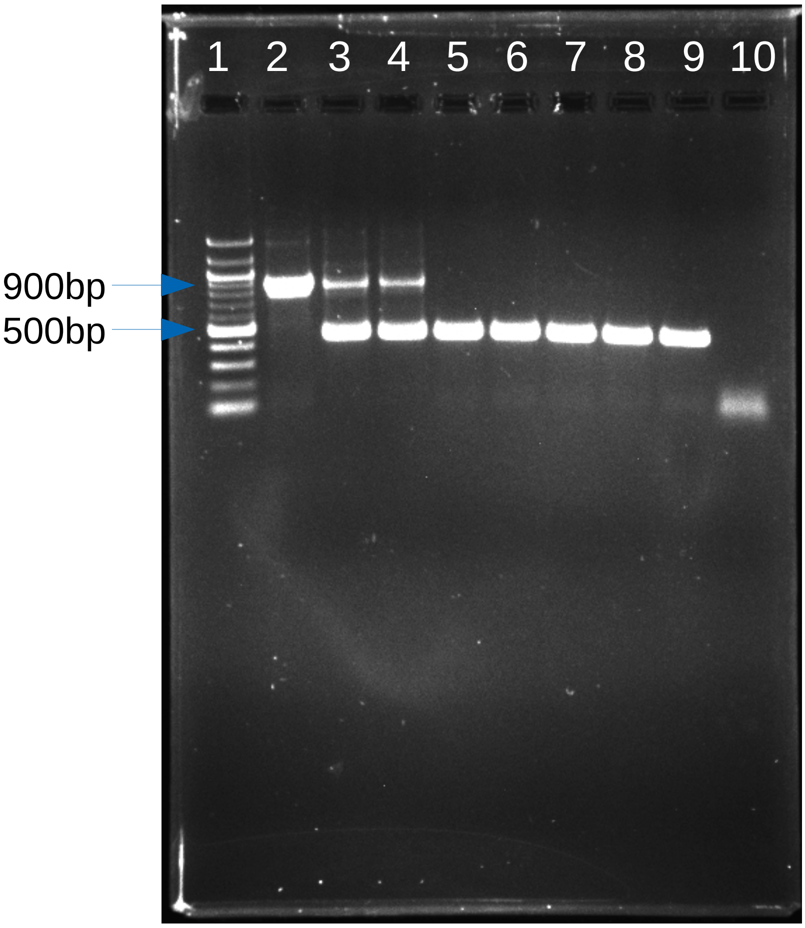

S4 Figure. Full-length 1.5% agarose gel electrophoresis showing representative results of the genotyping assay for Hereditary Myotonia in pigs. Well 1: Molecular-weight size marker. Well 2: Homozygous dominant control (868 bp amplicon - animal C1 of control group). Wells 3 and 4 : Heterozygous results (868 bp and 458 bp amplicons) respectively to animals F and G (G22 group). Wells 5 to 9: Homozygous recessive results (458 bp amplicon) respectively to animals A, B, C, D, and E (G22 group). Well 10: negative reaction control.
